# Supplementary material for: TIMELESS‐TIPIN and UBXN‐3 promote replisome disassembly during DNA replication termination in Caenorhabditis elegans
Source: EMBO J. 2021 Jul 16;40(17):e108053. doi: 10.15252/embj.2021108053 (PMC8408604; doi:10.15252/embj.2021108053)
Supplement: Supplementary file 2 — Appendix [file EMBJ-40-e108053-s001.pdf]

Table of Contents

Appendix Figure S1	Page 2
Appendix Figure S2	Page 3
Appendix Figure S3	Page 4
Appendix Figure S4	Page 5
Appendix Table S1	Page 6
Appendix Table S2	Page 17
Appendix Table S3	Page 20
Appendix Materials and Methods	Page 23

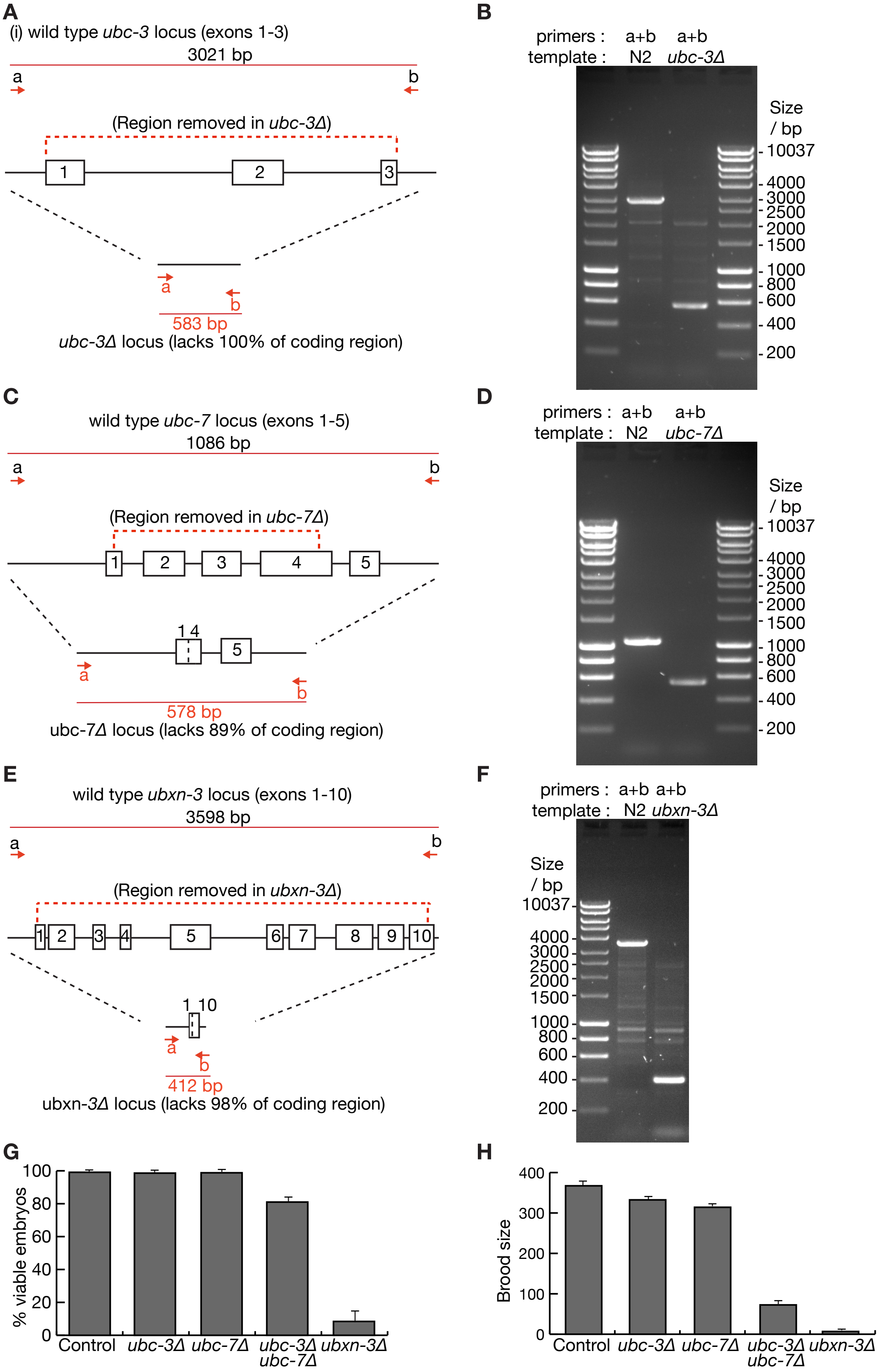

Deletion of the *C. elegans* genes *ubc-3*, *ubc-7* and *ubxn-3* by CRISPR-Cas9.
(**A**) Illustration of the region of the *C. elegans ubc-3* gene that was deleted by CRISPR-Cas9. Arrows indicate PCR primers that were used to monitor the deletion in adult worms. (**B**) PCR analysis of wt and *ubc-3Δ* worms with the indicated primers (a non-specific band is denoted with an asterisk). (**C**) Illustration of the region of the C. elegans *ubc-7* gene that was deleted by CRISPR-Cas9. Arrows indicate PCR primers that were used to monitor the deletion in adult worms. (**D**) PCR analysis of wt and *ubc-7Δ* worms with the indicated primers (a non-specific band is denoted with an asterisk). (**E**) Illustration of the region of the *C. elegans ubxn-3* gene that was deleted by CRISPR-Cas9. Arrows indicate PCR primers that were used to monitor the deletion in adult worms. (**F**) PCR analysis of wt and *ubxn-3Δ* worms with the indicated primers (a non-specific band is denoted with an asterisk). (**G**) The percentage of viable embryos was determined for the indicated genotypes as described in Materials & Methods. The data indicate the mean of three replicates with standard deviations. (**H**) The brood size of 10 worms of the indicated genotypes was determined as described in Materials & methods. The data present mean values with standard deviations.

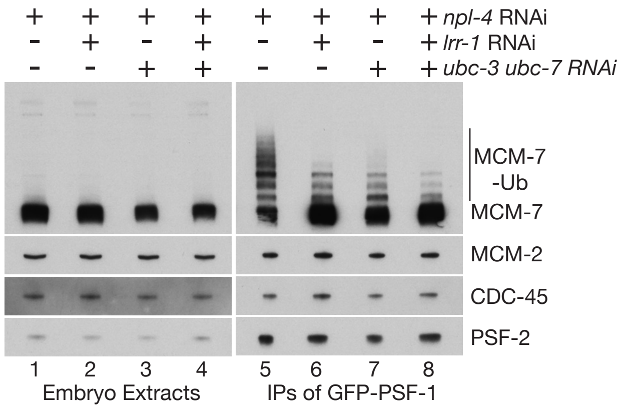

The *C. elegans* E2 enzymes UBC-3 and UBC-7 contribute to efficient ubiquitylation of CMG-MCM-7 by CUL-2^{LRR-1} during DNA replication termination.
In an analogous experiment to that described in Figure 1G, the ubiquitylation of CMG-MCM-7 was monitored after exposing worms to the indicated RNAi treatments.

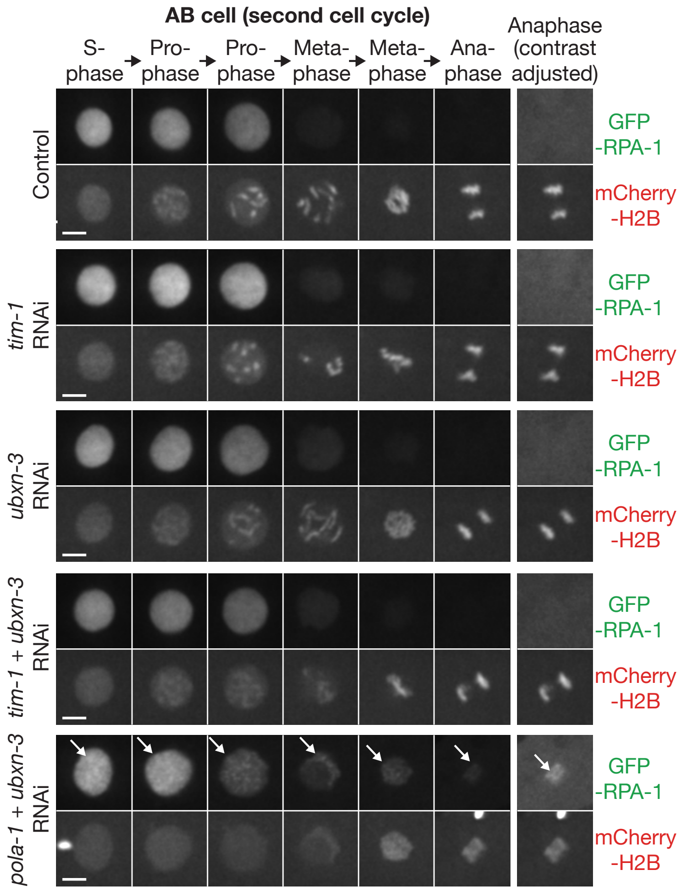

RNAi depletion of *tim-1* does not lead to accumulation of RPA-1 on chromatin.
The presence of GFP-RPA-1 on mitotic chromatin (indicated by white arrows) was monitored by spinning disk confocal microscopy (see Materials and Methods), in embryos derived from worms that also expressed mCherry-Histone H2B. The worms were fed on bacteria containing plasmids expressing the indicated RNAi ('Control' = empty vector). The scalebars correspond to 5 µm.

Xia et al, Appendix Figure S4

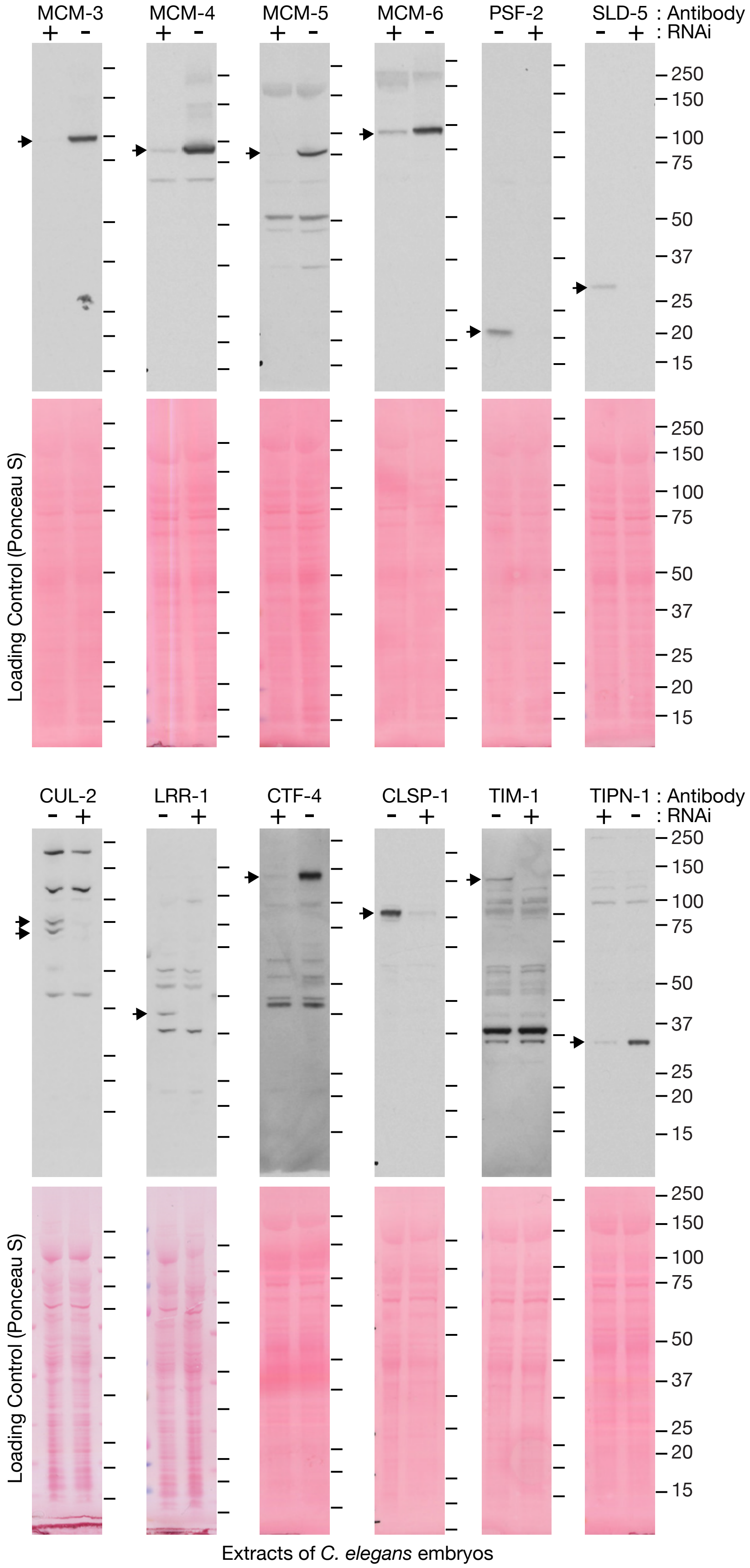

Validation of new antibodies that were generated in this study.
In each case, RNAi was used to deplete the corresponding *C. elegans* protein as indicated, before immunoblotting of embryonic extracts (upper panels). Ponceau S staining of the nitrocellulose membranes provided a loading control (lower panels).

Appendix Table S1

Reagents and resources from this study.

REAGENT or RESOURCE	SOURCE	IDENTIFIER
Antibodies		
Anti-MCM-2 [antigen 1-222 sheep]<br>Use 1 in 2,000 for immunoblotting	(Sonneville et al., 2017)	S750D
Anti-MCM-3 [antigen 1-222 sheep]<br>Use 1 in 1,000 for immunoblotting	This study, MRC PPU Reagents & Services	SA423
Anti-MCM-4 [antigen 1-222 sheep]<br>Use 1 in 1,000 for immunoblotting	This study, MRC PPU Reagents & Services	SA424
Anti-MCM-5 [antigen 1-222 sheep]<br>Use 1 in 3,000 for immunoblotting	This study, MRC PPU Reagents & Services	SA425
Anti-MCM-6[antigen 589-810 sheep]<br>Use 1 in 1,000 for immunoblotting	This study, MRC PPU Reagents & Services	SA417
Anti-MCM-7 [antigen 1-222 sheep]<br>Use 1 in 3,000 for immunoblotting	(Sonneville et al., 2017)	S797D
Anti-CDC-45 [antigen 1-222 sheep]<br>Use 1 in 500 for immunoblotting	(Sonneville et al., 2017)	S782D
Anti-PSF-1 [antigen full-length sheep]<br>Use 1 in 500 for immunoblotting	(Sonneville et al., 2017)	S789D
Anti-PSF-2 [antigen full-length sheep]<br>Use 1 in 1,500 for immunoblotting	This study, MRC PPU Reagents & Services	SA418
Anti-PSF-3 [antigen full-length rabbit]<br>Use 1 in 200 for immunoblotting	(Sonneville et al., 2017)	R3632
Anti-SLD-5 [antigen 1-222 sheep]<br>Use 1 in 1,500 for immunoblotting	This study, MRC PPU Reagents & Services	SA419
Anti-CLSP-1 [antigen 1-222 sheep]<br>Use 1 in 3,000 for immunoblotting	This study, MRC PPU Reagents & Services	SA426
Anti-CTF-4 [antigen 1-222 sheep]<br>Use 1 in 1,000 for immunoblotting	This study, MRC PPU Reagents & Services	SA416
Anti-TIM-1 [antigen 1-222 sheep]<br>Use 1 in 500 for immunoblotting	This study, MRC PPU Reagents & Services	SA430
Anti-TIPN-1 [antigen 1-222 sheep]<br>Use 1 in 3,000 for immunoblotting	This study, MRC PPU Reagents & Services	SA421
Anti-CUL-2 [antigen 569-790 sheep]<br>Use 1 in 250 for immunoblotting	This study, MRC PPU Reagents & Services	SA311
Anti-LRR-1 [antigen 1-222 sheep]<br>Use 1 in 250 for immunoblotting	This study, MRC PPU Reagents & Services	SA298
anti-sheep IgG HRP [from donkey]<br>Use 1 in 10,000 for immunoblotting	Sigma-Aldrich	A3415
anti-rabbit IgG HRP [from donkey]<br>Use 1 in 10,000 for immunoblotting	GE Healthcare	NA934
anti-mouse IgG HRP [from horse]<br>Use 1 in 10,000 for immunoblotting	Vector	PI-2000
FK2<br>Use 1 in 100 for immunoblotting	Enzo Life Sciences	BML-PW8810
Bacterial and Virus Strains		
Escherichia coli: Rosetta™ (DE3) pLysS cells: F^- *ompT* *hsdS$_B$*(r_B^- m_B^-) *gal dcm* (DE3) pLysSRARE (Cam^R)	Novagen	70956
Biological Samples		
Chemicals, Peptides, and Recombinant Proteins (unique isoforms unless mentioned otherwise)		

UBA-1, isoform a	This study	N/A
ULA-1_RFL-1	This study	N/A
UBC-12	This study	N/A
DCN-1, isoform a	This study	N/A
NED-8	This study	N/A
UBC-18	This study	N/A
ARI-1.1	This study	N/A
LET-70	This study	N/A
UBC-3, isoform a	This study	N/A
UBC-7	This study	N/A
UBC-1	This study	N/A
UBC-14	This study	N/A
CUL-2^{LRR-1} (including isoform b of CUL-2; other components only have one isoform)	This study	N/A
CUL-2-2R^{LRR-1} (including isoform b of CUL-2; other components only have one isoform)	This study	N/A
CUL-2^{VHL-1} (including isoform b of CUL-2; other components only have one isoform)	This study	N/A
CMG (including MCM-2 isoform a, MCM-4 isoform a, MCM-6 isoform a and SLD-5 isoform a; other components only have one isoform)	This study	N/A
TIM-1_TIPN-1	This study	N/A
CTF-18_RFC	This study	N/A
POL ε	This study	N/A
CLSP-1, isoform a	This study	N/A
CTF-4	This study	N/A
MCM-10	This study	N/A
Ubiquitin	Axel Knebel, MRC PPU Reagents & Services	DU20027
Ubiquitin (N-terminal FLAG)	Axel Knebel, MRC PPU Reagents & Services	DU46789
Ubiquitin [K0wt]	Axel Knebel, MRC PPU Reagents & Services	DU24363
Ubiquitin [K48R]	Axel Knebel, MRC PPU Reagents & Services	DU20042
Ubiquitin [K48wt]	Axel Knebel, MRC PPU Reagents & Services	DU24367
Ulp1 (403-621)	Alexander Stein	N/A
CDC-48.1	This study	N/A
UFD-1 (isoform a)_NPL-4.1(isoform a)	This study	N/A
UBXN-3, isoform b	This study	N/A
UBXN-3-ΔUBX (amino acids 1-530)	This study	N/A
Dynabeads M-270 Epoxy	ThermoFisher Scientific	14302D
Anti-FLAG M2 affinity gel	Sigma-Aldrich	A2220
Ni-NTA agarose	Qiagen	30210
Calmodulin sepharose 4B	GE Healthcare	17052901

IgG sepharose 6 Fast Flow	GE Healthcare	17096901
3Flag peptide	Sigma-Aldrich	F4799
GFP-Trap Agarose	Chromotek	gta-20
Roche cOmplete EDTA-free protease inhibitor cocktail	Roche	000000011873580001
Sigma protease inhibitor cocktail	Sigma-Aldrich	P8215
AEBSF	Sigma-Aldrich	A8456
Pepstatin A	Sigma-Aldrich	P5318
PreScission protease	Axel Knebel, MRC PPU Reagents & Services	DU34905
TEV protease	Axel Knebel, MRC PPU Reagents & Services	DU6811
Ubquitin PrG	Axel Knebel, MRC PPU Reagents & Services	DU49003
AcTEV	ThermoFisher Scientific	12575015
Critical Commercial Assays		
Experimental Models: Organisms/Strains		
C. elegans: Strain KAL1: *psf-1(lab1[gfp::TEV::S-tag::psf-1 + loxP unc-119(+) loxP])*	(Sonneville et al., 2017)	N/A
C. elegans: Strain KAL3: *psf-1(lab1); ltIs37[pie-1p::mCherry::his-58 + unc-119(+)]*	(Sonneville et al., 2017)	N/A
C. elegans: Strain KAL17: *psf-1(lab2[tap::psf-1 + loxP unc-119(+) loxP])*	This study, from InVivo Biosystems	Knu190
C. elegans: Strain KAL21: *ctf-4∆ (lab3 [3200 bp deletion])*	This study, from InVivo Biosystems	Knu468
C. elegans: Strain KAL55: *psf-1(lab2); ctf-4∆ (lab3)*	This study	N/A
C. elegans: Strain KAL90: *trul-1∆ (lab6 [3134 bp deletion])*	(Sonneville et al., 2019)	N/A
C. elegans: Strain KAL92: *psf-1(lab1); ltIs37; trul-1∆ (lab6)*	(Sonneville et al., 2019)	N/A
C. elegans: Strain KAL195: *ubc-3∆ (lab7 [2438 bp deletion])*	This study, from SunyBiotech	Syb2256
C. elegans: Strain KAL196: *ubc-7∆ (lab8 [508 bp deletion])*	This study, from SunyBiotech	Syb2285
C. elegans: Strain KAL198: *ubxn-3∆ (lab9 [3186 bp deletion])*	This study, from InVivo Biosystems	Knu580
S. cerevisiae: Strain *yJF1*: *MAT**a** ade2-1 ura3-1 his3-11,15 trp1-1 leu2-3,112 can1-100 bar1∆::hphNT pep4∆::kanMX*	(Frigola et al., 2013)	N/A
S. cerevisiae: Strain *YSS3*: *MAT**a** ade2-1 ura3-1 his3-11,15 trp1-1 leu2-3,112 can1-100 pep4∆::ADE2*	This study	N/A
S. cerevisiae: Strain *YSS4*: *MATα ade2-1 ura3-1 his3-11,15 trp1-1 leu2-3,112 can1-100 pep4∆::ADE2*	This study	N/A
S. cerevisiae: Strain *yYX1*(TIM-1_TIPN-1 purification): *MAT**a** ade2-1 ura3-1 his3-11,15 trp1-1 leu2-3,112 LEU2::pRS305-CBP-PreScissionScission-tipn-1-GAL1,10-tim-1 can1-100 pep4∆::ADE2*	This study	N/A

S. cerevisiae: Strain *yYX34*(MCM-2-7 expression): *MATα ade2-1 ura3-1 URA3::pRS306-mcm-2-GAL1,10-mcm-3 his3-11,15 trp1-1 TRP1::pRS304-mcm-6-GAL1,10-mcm-7 leu2-3,112 LEU2::pRS305-mcm-4-GAL1,10-mcm-5 can1-100 pep4Δ::ADE2*	This study	N/A
S. cerevisiae: Strain *yYX36*(GINS and CDC-45 expression): *MAT***a** *ade2-1 ura3-1 URA3::pRS306-TAP-psf-1-GAL1,10-sld-5 his3-11,15 HIS3::pRS303-GAL1,10-cdc-45 trp1-1 leu2-3,112 LEU2::pRS305-psf-3-GAL1,10-psf-2 can1-100 pep4Δ::ADE2*	This study	N/A
S. cerevisiae: Strain *yYX37*(CMG purification): *MATα / MAT***a** *ade2-1 / ade2-1 ura3-1 URA3::pRS306-mcm-2-GAL1,10-mcm-3 / ura3-1 URA3::pRS306-TAP-psf-1-GAL1,10-sld-5 his3-11,15 / his3-11,15 HIS3::pRS303-GAL1,10-cdc-45 trp1-1 TRP1::pRS304-mcm-6-GAL1,10-mcm-7 / trp1-1 leu2-3,112 LEU2::pRS305-mcm-4-GAL1,10-mcm-5 / leu2-3,112 LEU2::pRS305-psf-3-GAL1,10-psf-2 can1-100 / can1-100 pep4Δ::ADE2 / pep4Δ::ADE2*	This study	N/A
S. cerevisiae: Strain *yYX40*(CLSP-1 purification): *MAT***a** *ade2-1 ura3-1 his3-11,15 HIS3::pRS303-GAL1,10-clsp-1-TEV-5FLAG trp1-1 leu2-3,112 can1-100 pep4Δ::ADE2*	This study	N/A
S. cerevisiae: Strain *yYX41*(CTF-4 purification): *MAT***a** *ade2-1 ura3-1 his3-11,15 HIS-3::pRS303-GAL1,10-5FLAG-PreScissionScission-ctf-4 trp1-1 leu2-3,112 can1-100 pep4Δ::ADE2*	This study	N/A
S. cerevisiae: Strain *yYX42*(UBA-1 purification): *MAT***a** *ade2-1 ura3-1 his3-11,15 HIS3::pRS303-GAL1,10-ProteinA-TEV-uba-1 trp1-1 leu2-3,112 can1-100 pep4Δ::ADE2*	This study	N/A
S. cerevisiae: Strain *yYX43*(POLE purification): *MAT***a** *ade2-1 ura3-1 URA3::pRS306-pole-2-GAL1,10-pole-1 his3-11,15 trp1-1 leu2-3,112 LEU2::pRS305-pole-4-TEV-5FLAG-GAL1,10-pole-3 can1-100 pep4Δ::ADE2*	This study	N/A
S. cerevisiae: Strain *yYX44*(CUL-2^{LRR-1} purification): *MAT***a** *ade2-1 ura3-1 URA3::pRS306-cul-2-GAL1,10-rbx-1 his3-11,15 HIS3::pRS303-GAL1,10-lrr-1 trp1-1 leu2-3,112 LEU2::pRS305-TAP-elb-1-GAL1,10-elc-1 can1-100 pep4Δ::ADE2*	This study	N/A
S. cerevisiae: Strain *yYX46*(CTF-18 CTF-8 DSCC-1 expression): *MAT***a** *ade2-1 ura3-1 URA3::pRS306-dscc-1-GAL1,10-ctf-8 his3-11,15 HIS3::pRS303-GAL1,10-ProteinA-3TEV-ctf-18 trp1-1 leu2-3,112 can1-100 pep4Δ::ADE2*	This study	N/A
S. cerevisiae: Strain *yYX47*(RFC-2-5 expression): *MATα ade2-1 ura3-1 his3-11,15 trp1-1 TRP1::pRS304-rfc-4-GAL1,10-rfc-5 leu2-3,112 LEU2::pRS305-rfc-2-GAL1,10-rfc-3 can1-100 pep4Δ::ADE2*	This study	N/A

S. cerevisiae: Strain *yYX48*(CTF-18_RFC purification): *MAT***a** */ MATα ade2-1 / ade2-1 ura3-1 URA3::pRS306-dscc-1-GAL1,10-ctf-8 / ura3-1 his3-11,15 HIS3::pRS303-GAL1,10-ProteinA-3TEV-ctf-18 / his3-11,15 trp1-1 / trp1-1 TRP1::pRS304-rfc-4-GAL1,10-rfc-5 leu2-3,112 / leu2-3,112 LEU2::pRS305-rfc-2-GAL1,10-rfc-3 can1-100 / can1-100 pep4Δ::ADE2 / pep4Δ::ADE2*	This study	N/A
S. cerevisiae: Strain *yYX49*(CUL-2^{VHL-1} purification): *MAT***a** *ade2-1 ura3-1 URA3::pRS306-cul-2-GAL1,10-rbx-1 his3-11,15 HIS3::pRS303-GAL1,10-vhl-1 trp1-1 leu2-3,112 LEU2::pRS305-TAP-elb-1-GAL1,10-elc-1 can1-100 pep4Δ::ADE2*	This study	N/A
S. cerevisiae: Strain *yYX55*(CUL-2-2R^{LRR-1} purification): *MAT***a** *ade2-1 ura3-1 URA3::pRS306-cul-2-K719749R-GAL1,10-rbx-1 his3-11,15 HIS3::pRS303-GAL1,10-lrr-1 trp1-1 leu2-3,112 LEU2::pRS305-TAP-elb-1-GAL1,10-elc-1 can1-100 pep4Δ::ADE2*	This study	N/A
S. cerevisiae: Strain *yYX57*(ARI-1 purification): *MATa ade2-1 ura3-1 his3-11,15 HIS3::pRS303-GAL1,10-ProteinA-TEV-ari-1 trp1-1 leu2-3,112 can1-100 pep4Δ::ADE2*	This study	N/A
S. cerevisiae: Strain *yYX58*(ULA-1_RFL-1 purification): *MAT***a** *ade2-1 ura3-1 his3-11,15 HIS3::pRS303-rfl-1-GAL1,10-ProteinA-TEV-ula-1a trp1-1 leu2-3,112 can1-100 pep4Δ::ADE2*	This study	N/A
Oligonucleotides		
RNAi of *ubc-6* forward primer for construction of wRp-1: **7437**: gactagtATGAAGGAAGCCATGGAACTGCG	This study	N/A
RNAi of *ubc-6* reverse primer for construction of wRp-1: **7438**: CCGGGACCCAAATTCAGCATTTACAACTCGGAACCGTCTTTTG	This study	N/A
RNAi of *ubc-15* forward primer for construction of wRp-1: **7439**: ATGCTGAATTTGGGTCCCGGCGT	This study	N/A
RNAi of *ubc-15* reverse primer for construction of wRp-1: **7440**: GTAGATACTCCCGACGTCATTCGGATCTCTGAGCATCGGAACC	This study	N/A
RNAi of *ubc-26* forward primer for construction of wRp-1: **7441**: ATGACGTCGGGAGTATCTACAGT	This study	N/A
RNAi of *ubc-26* reverse primer for construction of wRp-1: **7442**: gactagtCTAAAAAGCCCAAATTTCCGGGC	This study	N/A
RNAi of *ubc-22* forward primer for construction of wRp-2: **7443**: gactagtATGAATGAAAACCAAAAAAGGTT	This study	N/A
RNAi of *ubc-22* reverse primer for construction of wRp-2: **7444**: AGAAAATTAACAACAGACATTTATTCTTGTGCCTGCTCGTCGC	This study	N/A
RNAi of *ubc-23* forward primer for construction of wRp-2: **7445**: ATGTCTGTTGTTAATTTTCTCAC	This study	N/A
RNAi of *ubc-23* reverse primer for construction of wRp-2: **7446**: AGTTTTCGAAGACACGCCATGTTTCTTATTCAAATTATCAGTA	This study	N/A

RNAi of *ubc-25* forward primer for construction of wRp-2: **7447**: ATGGCGTGTCTTCGAAAACTAAA	This study	N/A
RNAi of *ubc-25* reverse primer for construction of wRp-2: **7448**: gactagtTGCAACTCTCAATAGAGTAAGCT	This study	N/A
RNAi of *let-70* forward primer for construction of wRp-3: **7449**: gactagtATGGCTCTCAAAAGAATCCAGAA	This study	N/A
RNAi of *let-70* reverse primer for construction of wRp-3: **7450**: gactagtTCACATAGCGTACTTTTGCGTCC	This study	N/A
RNAi of *ubc-16* forward primer for construction of wRp-4: **7457**: gactagtATGTCAGATGCTGCGACAAGACG	This study	N/A
RNAi of *ubc-16* reverse primer for construction of wRp-4: **7458**: TATCCCTTAAATGAGGACTGTCAAACACTGTCGTCATGGAACC	This study	N/A
RNAi of *ubc-17* forward primer for construction of wRp-4: **7459**: CAGTCCTCATTTAAGGGATAGAA	This study	N/A
RNAi of *ubc-17* reverse primer for construction of wRp-4: **7460**: gactagtCTATGACGATGTTGATGGTTCAT	This study	N/A
RNAi of *ubc-1* forward primer for construction of wRp-5: **7461**: gactagtATGACGACGCCCAGCCGTAGACG	This study	N/A
RNAi of *ubc-1* reverse primer for construction of wRp-5: **7462**: GTTGACGCTTTTGAGTCCATTTAAGCATTCGATCCACTGGCTC	This study	N/A
RNAi of *ubc-3* forward primer for construction of wRp-5: **7463**: ATGGACTCAAAAGCGTCAACATC	This study	N/A
RNAi of *ubc-3* reverse primer for construction of wRp-5: **7464**: AGTAGGGAGGATTGCTCCATCTAATTTTCTCCTTGTCCCGAAT	This study	N/A
RNAi of *ubc-7* forward primer for construction of wRp-5: **7465**: ATGGAGCAATCCTCCCTACTTCT	This study	N/A
RNAi of *ubc-7* reverse primer for construction of wRp-5: **7466**: TTCAAAGCGTAACCAGCCATTCATTCTTCTTGACTTCTGCGAA	This study	N/A
RNAi of *ubc-14* forward primer for construction of wRp-5: **7467**: ATGGCTGGTTACGCTTTGAAGCG	This study	N/A
RNAi of *ubc-14* reverse primer for construction of wRp-5: **7468**: gactagtTTAGACTTCCGAAGCGGGAAGAC	This study	N/A
RNAi of *ubc-9* forward primer for construction of wRp-6: **7477**: gactagtATGTCGGGAATTGCTGCAGGACG	This study	N/A
RNAi of *ubc-9* reverse primer for construction of wRp-6: **7478**: gactagtCTACTCGAGCATTTGCTTCTGGA	This study	N/A
RNAi of *ubc-12* forward primer for construction of wRp-7: **7479**: gactagtATGTTCAATCTTCAAAAACGAAT	This study	N/A
RNAi of *ubc-12* reverse primer for construction of wRp-7: **7480**: gactagtTTAGCAATATCTAGAAATGTATT	This study	N/A
RNAi of *ubc-13* forward primer for construction of wRp-33: **7451**: gactagtATGGCCGGGCAACTTCCGCGTCG	This study	N/A
RNAi of *ubc-13* reverse primer for construction of wRp-33: **7452**: TTGAATGCTATGTTCGACATTCAGGCTTGAGCATAGTTCATCG	This study	N/A

RNAi of *ubc-20* forward primer for construction of wRp-33: **7453**: ATGTCGAACATAGCATTCAACAG	This study	N/A
RNAi of *ubc-20* reverse primer for construction of wRp-33: **7454**: TTCAATTTCGATACGCTCATTTAAGAGAAAATGTAGACCGTCG	This study	N/A
RNAi of *ubc-21* forward primer for construction of wRp-33: **7455**: ATGAGCGTATCGAAATTGAATAA	This study	N/A
RNAi of *ubc-21* reverse primer for construction of wRp-33: **7456**: gactagtCTAGTCAAAAATAAATTGAAGAG	This study	N/A
RNAi of *ubc-8* forward primer for construction of wRp-34: **7469**: gactagtATGACGTCAGCCACCGCAATCGG	This study	N/A
RNAi of *ubc-8* reverse primer for construction of wRp-34: **7470**: AGACGCCGTGTCGCTGACATCTAGATTGATGAAAATAAATATT	This study	N/A
RNAi of *ubc-18* forward primer for construction of wRp-34: **7471**: ATGTCAGCGACACGGCGTCTTCA	This study	N/A
RNAi of *ubc-18* reverse primer for construction of wRp-34: **7472**: TTTTCTTGATTTTCTTCCATCTATTCAGGCCGCTTTTCGGCGT	This study	N/A
RNAi of *ubc-19* forward primer for construction of wRp-34: **7562**: gccgctagcATGGAAGAAAATCAAGAAAAACT	This study	N/A
RNAi of *ubc-19* reverse primer for construction of wRp-34: **7474**: GCTGCTTGAAGTGTGCTCATTTAATTCAAATTCCCCAAATCCA	This study	N/A
RNAi of *ubc-1* reverse primer for construction of wRp-42: **7537**: gactagtTTAAGCATTCGATCCACTGGCTC	This study	N/A
RNAi of *ubc-3* forward primer for construction of wRp-43: **7538**: gactagtATGGACTCAAAAGCGTCAACATC	This study	N/A
RNAi of *ubc-3* reverse primer for construction of wRp-43: **7539**: gactagtCTAATTTTCTCCTTGTCCCGAAT	This study	N/A
RNAi of *ubc-7* forward primer for construction of wRp-44: **7540**: gactagtATGGAGCAATCCTCCCTACTTCT	This study	N/A
RNAi of *ubc-7* reverse primer for construction of wRp-44: **7541**: gactagtTCATTCTTCTTGACTTCTGCGAA	This study	N/A
RNAi of *ubc-14* forward primer for construction of wRp-45: **7542**: gactagtATGGCTGGTTACGCTTTGAAGCG	This study	N/A
RNAi of *tim-1* forward primer for construction of wRp-310: **7069**: gactagtAATGGTGAACCTGTGCCAACCTG	This study	N/A
RNAi of *tim-1* reverse primer for construction of wRp-310: **7070**: gactagtTTCCGTGAGTTGGACGTTATTCT	This study	N/A
RNAi of *ctf-4* forward primer for construction of wRp-316: **6485**: gactagtCTGCATTGATGCTTCAGGAGAAG	This study	N/A
RNAi of *ctf-4* reverse primer for construction of wRp-316: **6486**: gactagtGATTGAGTCGTCATCATCACTATC	This study	N/A
PCR check primer a of *ctf-4*Δ: **7109**: gctcaaagaagaggcgcttg	This study	N/A
PCR check primer b of *ctf-4*Δ: **7111**: CTCGTTTTCAGCTCTGCATC	This study	N/A
PCR check primer c of *ctf-4*Δ: **7110**: cagacacacgcgaagagggg	This study	N/A

PCR check primer a of *ubc-3*Δ: **9184**: CCGCGTGCACTATGCGTGTAACT	This study	N/A
PCR check primer b of *ubc-3*Δ: **9185**: AGTGCACTGTGATCAGCCTGTGT	This study	N/A
PCR check primer a of *ubc-7*Δ: **9187**: AACGAGTCCAACGCTTATGAGGA	This study	N/A
PCR check primer b of *ubc-7*Δ: **9188**: CTTCGTCGCCGCTATTTATCGAG	This study	N/A
PCR check primer a of *ubxn-3*Δ: **7487**: gtgtgtgtgtggggactcatat	This study	N/A
PCR check primer b of *ubxn-3*Δ: **7488**: gaggaacccgcatgtttttcaa	This study	N/A
mcm-10 forward primer for construction of pYXC53: **8953**: gaacagattggtggcATGGATCCCCTAGATGACCTAC	This study	N/A
mcm-10 reverse primer for construction of pYXC53: **8954**: gtgcggccgcttattaTCAGGAGCCAACAAACGGAC	This study	N/A
ubc-18 forward primer for construction of pYXC68: **9082**: gaacagattggtggcATGTCAGCGACACGGCGTCTTCAGAAGGAAC	This study	N/A
ubc-18 reverse primer for construction of pYXC68: **9083**: gtgcggccgcttattaCTATTCAGGCCGCTTTTCGGCGTGCTTTC	This study	N/A
ubc-12 forward primer for construction of pYXC72: **9111**: gaacagattggtggcATGTTCAATCTTCAAAAACGAATCAACGGCAAC	This study	N/A
ubc-12 reverse primer for construction of pYXC72: **9112**: gtgcggccgcttattaTTAGCAATATCTAGAAATGTATTCCCGAAC	This study	N/A
dcn-1a forward primer for construction of pYXC73: **9113**: gaacagattggtggcATGAATCGACTGAAGTCCGATCAAAAAAC	This study	N/A
dcn-1a reverse primer for construction of pYXC73: **9114**: gtgcggccgcttattaCTAATAATAACTTGGTGTCTCCATTTGCTG	This study	N/A
ned-8 forward primer for construction of pYXC74: **9115**: gaacagattggtggcATGCTCATCAAAGTTAAAACCTTGACTG	This study	N/A
ned-8 reverse primer for construction of pYXC74: **9116**: gtgcggccgcttattaTCCTCCGCGGAGAGCAAGAACCAGATGAAG	This study	N/A
cdc-48.1 forward primer for construction of pRF005: **7910**: gaacagattggtggcATGGCCTCGGTTCCAACGCATCAAAGCG	This study	N/A
cdc-48.1 reverse primer for construction of pRF005: **7911**: gtgcggccgcttattaGTTATAAAGGTCGTCATCATCCTGAGCAG	This study	N/A
ubxn-3 full-length forward primer for construction of pRF006: **7906**: gaacagattggtggcATGGATCTTACGGCTTCTTTGGAGGATG	This study	N/A

ubxn-3 full-length reverse primer for construction of pRF006: **7907**: gtgcggccgcttattaGATTTCCTCGACAAAAATCTGCTCCCTGG	This study	N/A
ufd-1 forward primer for construction of pRF007: **7914**: gaacagattggtggcATGCAAGCGTGGATACAACAAGGACTTC	This study	N/A
ufd-1 reverse primer for construction of pRF007: **7915**: gtgcggccgcttattaACGGGCTCCTCTCAGTGTTCGATTGCC	This study	N/A
npl-4.1 forward primer for construction of pRF008: **7916**: ATGGTACTTGAAGTCCCTCAAACTGAGC	This study	N/A
npl-4.1 forward primer for construction of pRF008: **7948**: gccTCTAGAAATAATTTTGTTTAACTTTAAGAAGGAGATATACCATGGTACTTGAAGTC	This study	N/A
npl-4.1 reverse primer for construction of pRF008: **7917**: gccctcgagTTAATCGGCAGCTGGCAATCCACACATC	This study	N/A
*5' oligo to make annealed substrate with 46bp double-strand DNA and 39nt of single-strand DNA (3' flap) TAGAGTAGGAAGTGATGGTAAGTGATTAGAGAATTGGAGAGTGTGTTTTTTTTTTTTTTTTTTTTTTTTTTTTTTTTTT*T*T*T*T*T [* denotes phosphorothioate bonds]*	Deegan et al., 2020	N/A
3' oligo to make annealed substrate with 46bp double-strand DNA and 39nt of single-strand DNA (3' flap) ACACACTCTCCAATTCTCTAATCACTTACCATCACTTCCTACTCTA	Deegan et al., 2020	N/A
ubxn-3-ΔUBX forward primer for construction of pRF099: **9317**: tattagatCTCAGCAAGTGGAGCACTCG	This study	N/A
ubxn-3-ΔUBX reverse primer for construction of pRF099: **9318**: ttgctgagATCTAATAAgcggccgcacatc	This study	N/A
Recombinant DNA		
pYXC1 [expresses TIM-1 and CBP-PreScission-TIPN-1, for TIM-1_TIPN-1 purification]	This study	DU70332
pYXC2 [expresses CUL-2b and RBX-1, for CUL-2^{LRR-1} purification]	This study	DU70333
pYXC14 [expresses MCM-2a and MCM-3, for CMG purification]	This study	DU70334
pYXC15 [expresses MCM-4a and MCM-5, for CMG purification]	This study	DU70335
pYXC17 [expresses CDC-45, for CMG purification]	This study	DU70336
pYXC19 [expresses PSF-3 and PSF-2, for CMG purification]	This study	DU70337
pYXC28 [expresses MCM-6a and MCM-7, for CMG purification]	This study	DU70338
pYXC29 [expresses TAP-PSF-1 and SLD-5a, for CMG purification]	This study	DU70339
pET15b 6HIS-TEV-UBC-3 [expresses 6HIS-TEV-UBC-3a, for UBC-3a purification]	This study; MRC PPU Reagents & Services	DU62204
pET15b 6HIS-TEV-UBC-7 [expresses 6HIS-TEV-UBC-7, for UBC-7 purification]	This study; MRC PPU Reagents & Services	DU62259
pYXC35 [expresses CLSP-1a-TEV-5FLAG, for CLSP-1a purification]	This study	DU70342
pYXC36 [expresses 5FLAG-PreScission-CTF-4, for CTF-4 purification]	This study	DU70343

pYXC37 [expresses PrA-3TEV-CTF-18, for CTF-18_RFC purification]	This study	DU70344
pYXC38 [expresses DSCC-1 and CTF-8, for CTF-18_RFC purification]	This study	DU70345
pYXC39 [expresses RFC-2 and RFC-3, for CTF-18_RFC purification]	This study	DU70346
pYXC40 [expresses RFC-4 and RFC-5, for CTF-18_RFC purification]	This study	DU70347
pYXC41 [expresses POLE-2 and POLE-1, for POL ε purification]	This study	DU70348
pYXC42 [expresses POLE-4-TEV-5FLAG and POLE-3, for POL ε purification]	This study	DU70349
pYXC43 [expresses PrA-TEV-UBA-1a, for UBA-1a purification]	This study	DU70350
pYXC44 [expresses LRR-1, for CUL-2^{LRR-1} purification]	This study	DU70351
pYXC45 [expresses VHL-1, for CUL-2^{VHL-1} purification]	This study	DU70352
pYXC47 [expresses TAP-ELB-1 and ELC-1, for CUL-2^{LRR-1} purification]	This study	DU70353
pET15b 6HIS-TEV-UBC-1 [expresses 6HIS-TEV-UBC-1, for UBC-1 purification]	This study; MRC PPU Reagents & Services	DU62228
pET15b 6HIS-TEV-UBC-14 [expresses 6HIS-TEV-UBC-14, for UBC-14 purification]	This study; MRC PPU Reagents & Services	DU62229
pET15b 6HIS-TEV-LET-70 [expresses 6HIS-TEV-LET-70, for LET-70 purification]	This study; MRC PPU Reagents & Services	DU62217
pYXC53 [expresses 14HIS-SUMO-MCM-10, for MCM-10 purification]	This study	DU70357
pYXC55 [expresses RFL-1 and PrA-TEV-ULA-1, for ULA-1_RFL-1 purification]	This study	DU70358
pYXC56 [expresses CUL-2-K719749R and RBX-1, for CUL-2-2R^{LRR-1} purification]	This study	DU70359
pYXC66 [expresses PrA-TEV-ARI-1, for ARI-1 purification]	This study	DU70360
pYXC68 [expresses 14HIS-SUMO-UBC-18, for UBC-18 purification]	This study	DU70361
pYXC72 [expresses 14HIS-SUMO-UBC-12, for UBC-12 purification]	This study	DU70362
pYXC73 [expresses 14HIS-SUMO-DCN-1a, for DCN-1a purification]	This study	DU70363
pYXC74 [expresses 14HIS-SUMO-NED-8, for NED-8 purification]	This study	DU70364
pRF005 [expresses 14HIS-SUMO-CDC-48.1, for CDC-48.1 purification]	This study	DU70365
pRF006 [expresses 14HIS-SUMO-UBXN-3b, for UBXN-3b purification]	This study	DU70366
pRF007 [expresses 14HIS-SUMO-UFD-1a, for UFD-1a purification]	This study	DU70367
pRF008 [expresses NPL-4.1a, for NPL-4.1a purification]	This study	DU70368
pRF099 [expresses 14HIS-SUMO-UBXN-3b-ΔUBX, for purification of UBXN-3-ΔUBX]	This study	DU70600
pFGET19-Ulp1(403-621)	Addgene	64697
L4440 [RNAi control plasmid]	(Sonneville et al., 2017)	DU70356
wRp-1 [RNAi of *ubc-6 ubc-15 ubc-26*]	This study	DU70369
wRp-2 [RNAi of *ubc-22 ubc-23 ubc-25*]	This study	DU70370

wRp-3 [RNAi of *let-70*]	This study	DU70371
wRp-4 [RNAi of *ubc-16 ubc-17*]	This study	DU70372
wRp-5 [RNAi of *ubc-1 ubc-3 ubc-7 ubc-14*]	This study	DU70373
wRp-6 [RNAi of *ubc-9*]	This study	DU70374
wRp-7 [RNAi of *ubc-12*]	This study	DU70375
wRp-30 [RNAi of *lrr-1*]	(Sonneville et al., 2017)	DU70376
wRp-32 [RNAi of *ubxn-3*]	(Sonneville et al., 2017)	DU70377
wRp-33 [[RNAi of *ubc-13 ubc-20 ubc-21*]	This study	DU70378
wRp-34 [RNAi of *ubc-8 ubc-18 ubc-19*]	This study	DU70379
wRp-42 [RNAi of *ubc-1*]	This study	DU70380
wRp-43 [RNAi of *ubc-3*]	This study	DU70381
wRp-44 [RNAi of *ubc-7*]	This study	DU70382
wRp-45 [RNAi of *ubc-14*]	This study	DU70383
wRp-54 [RNAi of *ubc-3 ubc-7*]	This study	DU70384
wRp-63 [RNAi of *lrr-1 ubc-3 ubc-7*]	This study	DU70385
wRp-64 [RNAi of *ubxn-3 ubc-3 ubc-7*]	This study	DU70386
wRp-68 [RNAi of *npl-4 lrr-1 ubc-3 ubc-7*]	This study	DU70387
wRp-69 [RNAi of *npl-4 ubc-3 ubc-7*]	This study	DU70388
wRp-310 [RNAi of *tim-1*]	This study	DU70389
wRp-311 [RNAi of *tipn-1*]	This study	DU70390
wRp-315 [RNAi of *tim-1 ubc-3 ubc-7*]	This study	DU70391
wRp-316 [RNAi of *npl-4 ctf-4*]	This study	DU70392
wRp-317 [RNAi of *npl-4 clsp-1*]	This study	DU70393
wRp-318 [RNAi of *npl-4 tim-1*]	This study	DU70394
wRp-319 [RNAi of *npl-4 tipn-1*]	This study	DU70395
wRp-330 [RNAi of *tim-1 ubxn-3*]	This study	DU70396
wRp-331 [RNAi of *tipn-1 ubxn-3*]	This study	DU70397
wRp-335 [RNAi of *lrr-1 ubxn-3*]	(Sonneville et al., 2017)	DU70398
wRp-336 [RNAi of *npl-4*]	(Sonneville et al., 2017)	DU70399
wRp-337 [RNAi of *npl-4 lrr-1*]	(Sonneville et al., 2017)	DU70400
Software and Algorithms		
ImageJ	National Institute of Health	https://imagej.nih.gov/ij/
Other		

Appendix Table S2

S. cerevisiae strains used in this study for protein expression.

Yeast strain	Protein expressed	Genotype	Notes
YJF1		*MAT***a** *ade2-1 ura3-1 his3-11,15 trp1-1 leu2-3,112 can1-100 bar1Δ::hphNT pep4Δ::kanMX*	
YSS3		*MAT***a** *ade2-1 ura3-1 his3-11,15 trp1-1 leu2-3,112 can1-100 pep4Δ::ADE2*	
YSS4		*MATα ade2-1 ura3-1 his3-11,15 trp1-1 leu2-3,112 can1-100 pep4Δ::ADE2*	
yYX1	TIM-1_CBP-PreScission-TIPN-1	*MAT***a** *ade2-1 ura3-1 his3-11,15 trp1-1 leu2-3,112 LEU2::pRS305-CBP-PreScission-tipn-1-GAL1,10-tim-1 can1-100 pep4Δ::ADE2*	based on YJF1
yYX34	MCM-2-7	*MATα ade2-1 ura3-1 URA3::pRS306-mcm-2-GAL1,10-mcm-3 his3-11,15 trp1-1 TRP1::pRS304-mcm-6-GAL1,10-mcm-7 leu2-3,112 LEU2::pRS305-mcm-4-GAL1,10-mcm-5 can1-100 pep4Δ::ADE2*	based on YSS4
yYX36	GINS (including ProteinA-3TEV-CBP-PreScission-PSF-1) + CDC-45	*MAT***a** *ade2-1 ura3-1 URA3::pRS306-TAP-psf-1-GAL1,10-sld-5 his3-11,15 HIS3::pRS303-GAL1,10-cdc-45 trp1-1 leu2-3,112 LEU2::pRS305-psf-3-GAL1,10-psf-2 can1-100 pep4Δ::ADE2*	based on YSS3
yYX37	Ce_CMG (including ProteinA-3TEV-CBP-PreScission-PSF-1)	*MATα / MAT***a** *ade2-1 / ade2-1 ura3-1 URA3::pRS306-mcm-2-GAL1,10-mcm-3 / ura3-1 URA3::pRS306-TAP-psf-1-GAL1,10-sld-5 his3-11,15 / his3-11,15 HIS3::pRS303-GAL1,10-cdc-45 trp1-1 TRP1::pRS304-mcm-6-GAL1,10-mcm-7 / trp1-1 leu2-3,112 LEU2::pRS305-mcm-4-GAL1,10-mcm-5 / leu2-3,112 LEU2::pRS305-psf-3-GAL1,10-psf-2 can1-100 / can1-100 pep4Δ::ADE2 / pep4Δ::ADE2*	mated from yYX34 and yYX36
yYX40	CLSP-1-TEV-5FLAG	*MAT***a** *ade2-1 ura3-1 his3-11,15 HIS3::pRS303-GAL1,10-clsp-1-*	based on YSS3

		TEV-5FLAG trp1-1 leu2-3,112 can1-100 pep4Δ::ADE2	
yYX41	5FLAG-PreScission-CTF-4	*MAT***a** *ade2-1 ura3-1 his3-11,15 HIS-3::pRS303-GAL1,10-5FLAG-PreScission-ctf-4 trp1-1 leu2-3,112 can1-100 pep4Δ::ADE2*	based on YSS3
yYX42	ProteinA-TEV-UBA-1	*MAT***a** *ade2-1 ura3-1 his3-11,15 HIS3::pRS303-GAL1,10-ProteinA-TEV-uba-1 trp1-1 leu2-3,112 can1-100 pep4Δ::ADE2*	based on YSS3
yYX43	POL ε (including POLE-4-TEV-5FLAG)	*MAT***a** *ade2-1 ura3-1 URA3::pRS306-pole-2-GAL1,10-pole-1 his3-11,15 trp1-1 leu2-3,112 LEU2::pRS305-pole-4-TEV-5FLAG-GAL1,10-pole-3 can1-100 pep4Δ::ADE2*	based on YSS3
yYX44	CUL-2[LRR-1](including ProteinA-3TEV-CBP-PreScission-ELB-1)	*MAT***a** *ade2-1 ura3-1 URA3::pRS306-cul-2-GAL1,10-rbx-1 his3-11,15 HIS3::pRS303-GAL1,10-lrr-1 trp1-1 leu2-3,112 LEU2::pRS305-TAP-elb-1-GAL1,10-elc-1 can1-100 pep4Δ::ADE2*	based on YSS3
yYX46	ProteinA-3TEV-CTF-18+CTF-8+DSCC-1	*MAT***a** *ade2-1 ura3-1 URA3::pRS306-dscc-1-GAL1,10-ctf-8 his3-11,15 HIS3::pRS303-GAL1,10-ProteinA-3TEV-ctf-18 trp1-1 leu2-3,112 can1-100 pep4Δ::ADE2*	based on YSS3
yYX47	RFC-2-5	*MAT*α *ade2-1 ura3-1 his3-11,15 trp1-1 TRP1::pRS304-rfc-4-GAL1,10-rfc-5 leu2-3,112 LEU2::pRS305-rfc-2-GAL1,10-rfc-3 can1-100 pep4Δ::ADE2*	based on YSS4
yYX48	CTF-18-RFC (including ProteinA-3TEV-CTF-18)	*MAT***a** / *MAT*α *ade2-1 / ade2-1 ura3-1 URA3::pRS306-dscc-1-GAL1,10-ctf-8 / ura3-1 his3-11,15 HIS3::pRS303-GAL1,10-ProteinA-3TEV-ctf-18 / his3-11,15 trp1-1 / trp1-1 TRP1::pRS304-rfc-4-GAL1,10-rfc-5 leu2-3,112 / leu2-3,112 LEU2::pRS305-rfc-2-GAL1,10-rfc-3 can1-100 / can1-100 pep4Δ::ADE2 / pep4Δ::ADE2*	mated from yYX46 and yYX47
yYX49	CUL-2[VHL-1] (including ProteinA-3TEV-CBP-PreScission-ELB-1)	*MAT***a** *ade2-1 ura3-1 URA3::pRS306-cul-2-GAL1,10-rbx-1 his3-11,15 HIS3::pRS303-GAL1,10-vhl-1 trp1-1 leu2-3,112*	based on YSS3

		LEU2::pRS305-TAP-elb-1-GAL1,10-elc-1 can1-100 pep4Δ::ADE2	
yYX55	CUL-2-2R^{LRR-1} (including ProteinA-3TEV-CBP-PreScission-ELB-1)	*MAT***a** *ade2-1 ura3-1 URA3::pRS306-cul-2-K719749R-GAL1,10-rbx-1 his3-11,15 HIS3::pRS303-GAL1,10-lrr-1 trp1-1 leu2-3,112 LEU2::pRS305-TAP-elb-1-GAL1,10-elc-1 can1-100 pep4Δ::ADE2*	based on YSS3
yYX57	ProteinA-TEV-ARI-1	*MAT***a** *ade2-1 ura3-1 his3-11,15 HIS3::pRS303-GAL1,10-ProteinA-TEV-ari-1 trp1-1 leu2-3,112 can1-100 pep4Δ::ADE2*	based on YSS3
yYX58	ProteinA-TEV-ULA-1_RFL-1	*MAT***a** *ade2-1 ura3-1 his3-11,15 HIS3::pRS303-rfl-1-GAL1,10-ProteinA-TEV-ula-1a trp1-1 leu2-3,112 can1-100 pep4Δ::ADE2*	based on YSS3

Appendix Table S3

Protein expression plasmids generated in this study. The codon usage of genes for expression in *S. cerevisiae* was optimised as described previously (Yeeles et al., 2015). The plasmids are available from MRC PPU Reagents and Services (https://mrcppureagents.dundee.ac.uk/).

Plasmid	Vector backbone & markers	Insert	Order number (MRC PPU Reagents & Services)	Expression system
pYXC1	pRS304 (TRP1; Amp^R)	*SacI - XmaI (CBP-PreScission-tipn-1-GAL1,10-tim-1)*	DU70332	*S. cerevisiae*
pYXC2	pRS306 (URA3; Amp^R)	*SacI - XmaI (cul-2 isoform b-GAL1,10-rbx-1)*	DU70333	*S. cerevisiae*
pYXC14	pRS306 (URA3; Amp^R)	*SacI - XmaI (mcm-2 isoform a-GAL1,10-mcm-3)*	DU70334	*S. cerevisiae*
pYXC15	pRS305 (LEU2; Amp^R)	*SacI - XmaI (mcm-4 isoform a-GAL1,10-mcm-5)*	DU70335	*S. cerevisiae*
pYXC17	pRS303 (HIS3; Amp^R)	*SacI - XmaI (GAL1,10-cdc-45)*	DU70336	*S. cerevisiae*
pYXC19	pRS305 (LEU2; Amp^R)	*SacI - XmaI (psf-3-GAL1,10-psf-2)*	DU70337	*S. cerevisiae*
pYXC28	pRS304 TRP1; Amp^R)	*SacI - XmaI (mcm-6 isoform a-GAL1,10-mcm-7)*	DU70338	*S. cerevisiae*
pYXC29	pRS306 (URA3; Amp^R)	*SacI - XmaI (TAP-psf-1-GAL1,10-sld-5 isoform a)*	DU70339	*S. cerevisiae*
pET15b 6HIS-TEV-UBC-3	pET15b (Amp^R)	*BamHI-NotI (6HIS-TEV-ubc-3 isoform a)*	DU62204	*E. coli*
pET15b 6HIS-TEV-UBC-7	pET15b (Amp^R)	*BamHI-NotI (6HIS-TEV-ubc-7)*	DU62259	*E. coli*
pYXC35	pRS303 (HIS3; Amp^R)	*SacI - XmaI (GAL1,10-clsp-1 isoform a-TEV-5FLAG)*	DU70342	*S. cerevisiae*
pYXC36	pRS303 (HIS3; Amp^R)	*SacI - XmaI (GAL1,10-5FLAG-PreScission-ctf-4)*	DU70343	*S. cerevisiae*
pYXC37	pRS303	*SacI - XmaI*	DU70344	*S. cerevisiae*

	(HIS3; Amp^R)	*(GAL1,10-ProteinA-3TEV-ctf-18)*		
pYXC38	pRS306 (URA3; Amp^R)	*SacI - XmaI (dscc-1-GAL1,10-ctf-8)*	DU70345	*S. cerevisiae*
pYXC39	pRS305 (LEU2; Amp^R)	*SacI - XmaI (rfc-2-GAL1,10-rfc-3)*	DU70346	*S. cerevisiae*
pYXC40	pRS304 TRP1; Amp^R)	*SacI - XmaI (rfc-4-GAL1,10-rfc-5)*	DU70347	*S. cerevisiae*
pYXC41	pRS306 (URA3; Amp^R)	*SacI - XmaI (pole-2-GAL1,10-pole-1)*	DU70348	*S. cerevisiae*
pYXC42	pRS305 (LEU2; Amp^R)	*SacI - XmaI (pole-4-TEV-5FLAG-GAL1,10-pole-3)*	DU70349	*S. cerevisiae*
pYXC43	pRS303 (HIS3; Amp^R)	*SacI - XmaI (GAL1,10-ProteinA-TEV-uba-1 isoform a)*	DU70350	*S. cerevisiae*
pYXC44	pRS303 (HIS3; Amp^R)	*SacI - XmaI (GAL1,10-lrr-1)*	DU70351	*S. cerevisiae*
pYXC45	pRS303 (HIS3; Amp^R)	*SacI - XmaI (GAL1,10-vhl-1)*	DU70352	*S. cerevisiae*
pYXC47	pRS305 (LEU2; Amp^R)	*SacI - XmaI (TAP-elb-1-GAL1,10-elc-1)*	DU70353	*S. cerevisiae*
pET15b 6HIS-TEV-UBC-1	pET15b (Amp^R)	*BamHI-NotI (6HIS-TEV-ubc-1)*	DU62228	*E. coli*
pET15b 6HIS-TEV-UBC-14	pET15b (Amp^R)	*BamHI-NotI (6HIS-TEV-ubc-14)*	DU62229	*E. coli*
pET15b 6HIS-TEV-LET-70	pET15b (Amp^R)	*BamHI-NotI (6HIS-TEV-let-70)*	DU62217	*E. coli*
pYXC53	pK27SUMO (Kan^R)	*14HIS-SUMO-mcm-10 (PCR & Gibson cloning; oligos 8593+8954)*	DU70357	*E. coli*
pYXC55	pRS303 (HIS3; Amp^R)	*SacI - XmaI (rfl-1-GAL1,10-ProteinA-TEV-ula-1[a])*	DU70358	*S. cerevisiae*
pYXC56	pRS306 (URA3; Amp^R)	*SacI - XmaI (cul-2-K719749R-GAL1,10-rbx-1)*	DU70359	*S. cerevisiae*
pYXC66	pRS303	*SacI - XmaI*	DU70360	*S. cerevisiae*

	(HIS3; Amp^R)	*(GAL1,10-ProteinA-TEV-ari-1)*		
pYXC68	pK27SUMO (Kan^R)	*14HIS-SUMO-ubc-18 (PCR & Gibson cloning; oligos 9082+9083)*	DU70361	*E. coli*
pYXC72	pK27SUMO (Kan^R)	*14HIS-SUMO-ubc-12 (PCR & Gibson cloning; oligos 9011+9012)*	DU70362	*E. coli*
pYXC73	pK27SUMO (Kan^R)	*14HIS-SUMO-dcn-1 isoform a (PCR & Gibson cloning; oligos 9013+9014)*	DU70363	*E. coli*
pYXC74	pK27SUMO (Kan^R)	*14HIS-SUMO-ned-8 (PCR & Gibson cloning; oligos 9015+9016)*	DU70364	*E. coli*
pRF005	pK27SUMO (Kan^R)	*14HIS-SUMO-cdc-48.1 (PCR & Gibson cloning; oligos 7910+7911)*	DU70365	*E. coli*
pRF006	pK27SUMO (Kan^R)	*14HIS-SUMO-ubxn-3 isoform b (PCR & Gibson cloning; oligos 7906+7907)*	DU70366	*E. coli*
pRF007	pK27SUMO (Kan^R)	*14HIS-SUMO-ufd-1 isoform a (PCR & Gibson cloning; oligos 7914+7915)*	DU70367	*E. coli*
pRF008	pET28c (Kan^R)	*npl-4.1 isoform a (PCR & Gibson cloning; oligos 7948+7917)*	DU70368	*E. coli*
pRF099	pK27SUMO (Kan^R)	*14HIS-SUMO-ubxn-3-ΔUBX (PCR from pRF006 by oligos 9317+9318)*	DU70600	*E. coli*

Appendix Materials and Methods

Protein expression in budding yeast cells:

CLSP-1

Yeast cell powder was thawed in lysis buffer (buffer A / 0.5 M NaCl / 1X protease inhibitor cocktail 2). The mixture was centrifuged at 100,000 x g at 4°C for 0.5 h, followed by another step of centrifugation at 235,000 x g at 4°C for 1 h. After spinning, the soluble extract was recovered and mixed with 3 ml anti-FLAG M2 affinity gel. The mixture was incubated at 4 °C for 2 h with rotation.

Resin was collected and washed extensively with buffer A / 0.5 M NaCl / 1X protease inhibitor cocktail 2. Then resin was incubated with 10ml buffer A / 0.5 M NaCl / 1X protease inhibitor cocktail 2 / 10 mM $Mg(OAc)_2$ / 2 mM ATP at 4 °C for 10 minutes to remove chaperones, then washed extensively with buffer A / 0.5 M NaCl. CLSP-1 was eluted in 1 column volume of buffer A / 0.5 M NaCl / 0.5 mg / ml 3FLAG peptide, then 1 column volume of buffer A / 0.5 M NaCl / 0.25 mg / ml 3FLAG peptide twice.

The eluate fractions were diluted to 0.15 M NaCl and loaded onto a 1 ml HiTrap Q column in buffer E / 0.15 M NaCl. CLSP-1 was then eluted with a 20 ml gradient from 0.15 – 1 M NaCl in buffer A. The peak fractions were pooled, diluted to 0.15 M NaCl and loaded onto a 1 ml HiTrap Q column in buffer E / 0.15 M NaCl. CLSP-1 was then eluted with a 10 ml gradient from 0.3 – 0.6 M NaCl in buffer A.

Peak fractions containing CLSP-1 were pooled and dialyzed against buffer G / 0.3 M KOAc at 4 °C for overnight. The dialyzed sample was recovered, aliquoted, snap frozen in liquid nitrogen and stored at – 80 °C.

CMG helicase

Yeast cell powder was thawed in buffer E / 0.2 M KCl / 2 mM $Mg(OAc)_2$ / 1X protease inhibitor cocktail 2. Universal Nuclease (Pierce™, 88702, ThermoFisher Scientific) was then added to 250 U / ml to the whole cell extract and the sample was incubated at 4 °C for 0.5 h with rotation. The mixture was centrifuged at 100,000 x g at 4°C for 0.5 h, followed by another step of centrifugation at 235,000 x g at 4°C for 1 h. After spinning, the soluble extract was recovered and mixed with 2 ml IgG resin (17096901, GE). The mixture was incubated at 4 °C for 2 h with rotation.

Resin was collected and washed extensively with buffer E / 0.2 M KCl / 2 mM $Mg(OAc)_2$ / 1X protease inhibitor cocktail 2. The resin was then incubated with 10ml buffer E / 0.2 M KCl / 10 mM $Mg(OAc)_2$ / 1X protease inhibitor cocktail 2 / 2 mM ATP at 4 °C for 10 minutes to remove chaperones, then washed extensively with buffer E / 0.2 M KCl / 2 mM $Mg(OAc)_2$. The purified proteins were then eluted by overnight incubation with rotation in 2 ml buffer E / 0.2 M KCl / 2 mM $Mg(OAc)_2$ containing 100 μg TEV protease.

The supernatant was collected, and the resin was further eluted twice with 2ml buffer E / 0.2 M KCl / 2 mM $Mg(OAc)_2$. The pooled eluate was diluted to 10 ml and loaded onto a 1 ml HiTrap Q column in buffer E / 0.2 M KCl / 2 mM $Mg(OAc)_2$. CMG was eluted with a 20 ml gradient from 0.2 – 1 M KCl in buffer E / 2 mM $Mg(OAc)_2$. The peak fractions were pooled, diluted to 10 ml and loaded onto a 0.2 ml Mini Q column in buffer E / 0.2 M KCl / 2 mM $Mg(OAc)_2$. Subsequently, CMG was eluted with a 4 ml gradient from 0.2 – 0.6 M KCl in buffer E / 2 mM $Mg(OAc)_2$. The CMG containing fractions were then pooled and loaded onto a 24 ml Superose 6 column in buffer E / 0.2 M KCl / 2 mM $Mg(OAc)_2$. Peak fractions containing CMG were pooled and re-loaded onto a 0.2 ml MiniQ column in buffer E / 0.2 M KCl / 2

mM $Mg(OAc)_2$. Finally, CMG was eluted with a 2.5 ml gradient from 0.2 – 0.6 M KCl in buffer E / 2 mM $Mg(OAc)_2$.

Peak fractions containing CMG were pooled and dialyzed against buffer E / 0.3 M KOAc / 2 mM $Mg(OAc)_2$ at 4 °C overnight. The dialyzed sample was recovered, aliquoted, snap frozen in liquid nitrogen and stored at – 80 °C.

CTF-4

Yeast cell powder was thawed in buffer A / 0.5 M NaCl / 1X protease inhibitor cocktail 2. Universal Nuclease (Pierce™, 88702, ThermoFisher Scientific) was then added to 250 U / ml to the whole cell extract and the sample was incubated at 4 °C for 0.5 h with rotation. The mixture was centrifuged at 100,000 x g at 4°C for 0.5 h, followed by another step of centrifugation at 235,000 x g at 4°C for 1 h. After spinning, the soluble extract was recovered and mixed with 3 ml anti-FLAG M2 affinity gel (Sigma-Aldrich, A2220). The mixture was incubated at 4 °C for 2 h with rotation.

Resin was collected and washed extensively with buffer A / 0.5 M NaCl / 1X protease inhibitor cocktail 2. The resin was then incubated with 10ml buffer A / 0.5 M NaCl / 1X protease inhibitor cocktail 2 / 10 mM $Mg(OAc)_2$ / 2 mM ATP at 4 °C for 10 minutes to remove chaperones and washed extensively with buffer A / 0.5 M NaCl. CTF-4 was eluted in 1 column volume of buffer A / 0.5 M NaCl / 0.5 mg / ml 3FLAG peptide, then 1 column volume of buffer A / 0.5 M NaCl / 0.25 mg / ml 3FLAG peptide twice. PreScission protease (100 μg) was added to the eluate fractions and the mixture was incubated at 4 °C overnight with rotation.

The sample was diluted to 0.2 M NaCl and loaded onto a 1 ml HiTrap Q column in buffer A / 0.2 M NaCl. CTF-4 was eluted with a 20 ml gradient from 0.2 –

1 M NaCl in buffer A. The peak fractions were pooled and mixed with 0.5 ml anti-FLAG M2 affinity gel to remove any uncleaved protein. The mixture was incubated at 4 °C for 1 h with rotation. The flow-through fraction was collected and diluted to 0.2 M NaCl. The sample was loaded onto a 1 ml HiTrap Q column in buffer A / 0.2 M NaCl. CTF-4 was eluted with a 15 ml gradient from 0.2 – 0.5 M NaCl in buffer A. The peak fractions were pooled, diluted to 0.2 M NaCl and loaded onto a 1 ml HiTrap Q column in buffer A / 0.2 M NaCl. CTF-4 was eluted with a 5 ml gradient from 0.2 – 0.5 M NaCl in buffer A. The peak fractions containing CTF-4 were pooled, aliquoted, snap frozen in liquid nitrogen and stored at – 80 °C.

CTF-18_RFC

Yeast cell powder was thawed in buffer F / 0.5 M NaCl / 1X protease inhibitor cocktail 2. Universal Nuclease (Pierce™, 88702, ThermoFisher Scientific) was then added to 250 U / ml to the whole cell extract and the sample was incubated at 4 °C for 0.5 h with rotation. The mixture was centrifuged at 100,000 x g at 4°C for 0.5 h, followed by another step of centrifugation at 235,000 x g at 4°C for 1 h. After spinning, the soluble extract was recovered and mixed with 2 ml IgG resin (17096901, GE). The mixture was incubated at 4 °C for 2 h with rotation.

Resin was collected and washed extensively with buffer F / 0.5 M NaCl / 1X protease inhibitor cocktail 2. The resin was then incubated with 10ml buffer F / 0.5 M NaCl / 1X protease inhibitor cocktail 2 / 10 mM $Mg(OAc)_2$ / 2 mM ATP at 4 °C for 10 minutes to remove chaperones, then washed extensively with buffer F / 0.5 M NaCl. The purified proteins were then eluted by overnight incubation with rotation in 2 ml buffer F / 0.5 M NaCl containing 100 μg TEV protease.

The supernatant was collected, and the resin was further eluted twice with 2ml buffer F / 0.5 M NaCl. The pooled eluate was concentrated and loaded onto a 24 ml Superose 6 column in buffer F / 0.5 M NaCl. The peak fractions were pooled, concentrated, aliquoted, snap frozen in liquid nitrogen and stored at – 80 °C.

$CUL\text{-}2^{LRR\text{-}1}$ and $CUL\text{-}2\text{-}2R^{LRR\text{-}1}$* (CUL-2-2R contains *K719R and K749R mutations)

Yeast cell powder was thawed in buffer B / 0.5 M NaCl / 1X protease inhibitor cocktail 2. The mixture was centrifuged at 100,000 x g at 4°C for 0.5 h, followed by another step of centrifugation at 235,000 x g at 4°C for 1 h. After spinning, the soluble extract was recovered and mixed with 2 ml IgG resin (17096901, GE). The mixture was incubated at 4 °C for 2 h with rotation.

Resin was collected and washed extensively with buffer B / 0.5 M NaCl / 1X protease inhibitor cocktail 2. Then resin was incubated with 20 ml buffer B / 0.5 M NaCl / 1X protease inhibitor cocktail 2 / 10 mM $Mg(OAc)_2$ / 2 mM ATP at 4 °C for 10 minutes to remove chaperones, then washed extensively with buffer B / 0.5 M NaCl. The purified proteins were then eluted by overnight incubation with rotation in 2 ml buffer B / 0.5 M NaCl containing 100 μg TEV protease.

The supernatant was collected, and the resin was further eluted twice with 2ml buffer B / 0.5 M NaCl. The pooled eluate was concentrated and loaded onto a 24 ml Superdex 200 column in buffer B / 0.5 M NaCl. The peak fractions were pooled, concentrated and re-loaded onto a 24 ml Superdex 200 column in buffer B / 0.5 M NaCl. Subsequently, the peak fractions were pooled, concentrated, aliquoted, snap frozen in liquid nitrogen and stored at – 80 °C.

$CUL\text{-}2^{VHL\text{-}1}$

Yeast cell powder was thawed in buffer B / 0.3 M KOAc / 1X protease inhibitor cocktail 2. The mixture was centrifuged at 100,000 x g at 4°C for 0.5 h, followed by another step of centrifugation at 235,000 x g at 4°C for 1 h. After spinning, the soluble extract was recovered and mixed with 2 ml IgG resin (17096901, GE). The mixture was incubated at 4 °C for 2 h with rotation. The resin was then collected and washed extensively with buffer B / 0.3 M KOAc / 1X protease inhibitor cocktail 2. Subsequently, the resin was incubated with 10ml buffer B / 0.3 M KOAc / 1X protease inhibitor cocktail 2 / 10 mM $Mg(OAc)_2$ / 2 mM ATP at 4 °C for 10 minutes to remove chaperones, then washed extensively with buffer B / 0.3 M KOAc. The purified proteins were then eluted by overnight incubation with rotation in 2 ml buffer B / 0.3 M KOAc containing 100 μg TEV protease.

The supernatant was collected, and the resin was further eluted twice with 2ml buffer B / 0.3 M KOAc. The pooled eluate was concentrated and loaded onto a 24 ml Superdex 200 column in buffer B / 0.3 M KOAc. The peak fractions were pooled, concentrated, aliquoted, snap frozen in liquid nitrogen and stored at – 80 °C.

DNA polymerase epsilon

Yeast cell powder was thawed in buffer A / 0.3 M NaCl / 1X protease inhibitor cocktail 2. Universal Nuclease (Pierce™, 88702, ThermoFisher Scientific) was then added to 250 U / ml to the whole cell extract and the sample was incubated at 4 °C for 0.5 h with rotation. The mixture was centrifuged at 100,000 x g at 4°C for 0.5 h, followed by another step of centrifugation at 235,000 x g at 4°C for 1 h. After spinning, the soluble extract was recovered and mixed with 3 ml anti-FLAG M2 affinity gel (A2220, Sigma-Aldrich). The mixture was incubated at 4 °C for 2 h with rotation.

Resin was collected and washed extensively with buffer A / 0.3 M NaCl / 1X protease inhibitor cocktail 2. The resin was then incubated with 10ml buffer A / 0.3 M NaCl / 1X protease inhibitor cocktail 2 / 10 mM $Mg(OAc)_2$ + 2 mM ATP at 4 °C for 10 minutes to remove chaperones, then washed extensively with buffer A / 0.3 M NaCl. The purified proteins were then eluted by overnight incubation with rotation in 3 ml buffer A / 0.3 M NaCl containing 100 μg TEV protease.

The supernatant was collected, and the resin was eluted twice with 2ml buffer A / 0.3 M NaCl. The pooled eluate was diluted to 10 ml and loaded onto a 1 ml HiTrap Heparin column in buffer A/ 0.3 M NaCl. POLε was eluted with a 20 ml gradient from 0.3 – 1 M NaCl in buffer A. The peak fractions were pooled, concentrated and loaded onto a 24 ml Superose 6 column in buffer A / 0.3 M NaCl. The peak fractions containing POLε were pooled, concentrated, aliquoted, snap frozen in liquid nitrogen and stored at – 80 °C.

TIM-1_TIPN-1

Yeast cell powder was thawed in buffer A / 0.2 M NaCl / 1X protease inhibitor cocktail 2. The mixture was centrifuged at 100,000 x g at 4°C for 0.5 h, followed by another step of centrifugation at 235,000 x g at 4°C for 1 h. After spinning, the soluble extract was supplemented with 2 mM $CaCl_2$, 2 ml calmodulin affinity resin (17052901, GE) was added, and the mixture incubated with rotation at 4 °C for 2 h.

Resin was collected and washed extensively with buffer A / 0.2 M NaCl / 2mM $CaCl_2$ / 1X protease inhibitor cocktail 2. Then the resin was incubated with 10ml buffer A / 0.2 M NaCl / 2mM $CaCl_2$ / 1X protease inhibitor cocktail 2 / 10 mM $Mg(OAc)_2$ / 2 mM ATP at 4 °C for 10 minutes to remove chaperones, then washed extensively with buffer A / 0.2 M NaCl / 2mM $CaCl_2$. TIM-1_TIPN-1 was eluted in

buffer A / 0.2 M NaCl / 2mM EDTA / 2mM EGTA. The purified proteins were then eluted addition of 100 μg PreScission protease to the eluate fraction, followed by overnight incubation with rotation at 4 °C.

The sample was concentrated and loaded onto a 24 ml Superdex 200 column in buffer A / 0.3 M KOAc. The peak fractions were pooled, concentrated and re-loaded onto a 24 ml Superdex 200 column in buffer A / 0.3 M KOAc. The peak fractions were pooled, concentrated, aliquoted, snap frozen in liquid nitrogen and stored at – 80 °C.

UBA-1, ULA-1_RFL-1 and ARI-1.1

Yeast cell powder was thawed in buffer A / 0.5 M NaCl / 1X protease inhibitor cocktail 2. The mixture was centrifuged at 100,000 x g at 4°C for 0.5 h, followed by another step of centrifugation at 235,000 x g at 4°C for 1 h. After spinning, the soluble extract was recovered and mixed with 2 ml IgG resin (17096901, GE). The mixture was incubated at 4 °C for 2 h with rotation.

The resin was then collected and washed extensively with buffer A / 0.5 M NaCl / 1X protease inhibitor cocktail 2. The resin was then incubated with 10 ml buffer A / 0.5 M NaCl / 1X protease inhibitor cocktail 2 / 10 mM $Mg(OAc)_2$ / 2 mM ATP at 4 °C for 10 minutes to remove chaperones, then washed extensively with buffer A / 0.5 M NaCl. The purified proteins were then eluted by overnight incubation with rotation in 2 ml buffer A / 0.5 M NaCl containing 100 μg TEV protease.

Subsequently, the supernatant was collected, and the resin was further eluted twice with 2ml buffer A / 0.5 M NaCl. For UBA-1, the pooled eluate was concentrated and loaded onto a 24 ml Superdex 200 column in buffer A / 0.3 M KOAc. The peak fractions containing UBA-1 were pooled, concentrated, aliquoted

and snap frozen in liquid nitrogen. For ULA-1_RFL-1 and ARI-1.1, the supernatant fractions were concentrated and loaded onto a 24 ml Superdex 200 column in buffer A / 0.5 M NaCl. The peak fractions containing ULA-1_RFL-1 or ARI-1.1 were pooled, concentrated, aliquoted, snap frozen in liquid nitrogen and then stored at -80 °C.

Protein expression in *E. coli*:

CDC-48.1

Rosetta cells were transformed with the CDC-48.1 expression vectors. For induction, 1 mM IPTG was added and cells were incubated at 24 °C for 16 h. The cell pellets were resuspended in 20 ml of buffer H / 0.5 M NaCl / 40 mM imidazole / 5 mM $Mg(OAc)_2$ / 0.1mM ATP / 1X protease inhibitor cocktail 3 with 500 μg / ml Lysozyme, then the mixture was incubated at 4 °C for 0.5 h with rotation. Subsequently, the sample was sonicated twice for 90 s (15 s on, 30 s off) at 40% on a Branson Digital Sonifier. The mixture was then centrifuged at 100,000 x g at 4°C for 0.5 h. After spinning, the soluble extract was recovered and mixed with 2 ml Ni-NTA resin (30210, QIAGEN). The mixture was incubated at 4 °C for 2 h with rotation.

Resin was collected and washed extensively with buffer H / 0.5 M NaCl / 40 mM imidazole / 5 mM $Mg(OAc)_2$ / 0.1mM ATP. Proteins were eluted with 6 ml of buffer H / 0.5 M NaCl / 0.5 M imidazole / 5 mM $Mg(OAc)_2$ / 0.1mM ATP. Ulp1 protease (10 μg/ml) was added and the mixture was incubated for 1 h on ice. The sample was then concentrated to 500 μl and loaded onto a 24 ml Superose 6 column in 20 mM Hepes-KOH (pH 7.4), 0.3 M sorbitol, 0.15 M NaCl, 5 mM

$Mg(OAc)_2$, 0.1 mM ATP, 0.5 mM TCEP. Finally, CDC-48.1-containing fractions were pooled, concentrated, aliquoted, snap frozen in liquid nitrogen and stored at – 80 °C.

DCN-1a

Pellets were resuspended in 20 ml of buffer A / 0.5 M NaCl / 40 mM imidazole / 1X protease inhibitor cocktail 3 with 500 μg / ml Lysozyme, then the mixture was incubated at 4 °C for 0.5 h with rotation. Subsequently, the sample was sonicated twice for 90 s (15 s on, 30 s off) at 40% on a Branson Digital Sonifier. The mixture was centrifuged at 100,000 x g at 4°C for 0.5 h. After spinning, the soluble extract was recovered and mixed with 1 ml Ni-NTA resin (30210, QIAGEN). The mixture was incubated at 4 °C for 2 h with rotation.

The resin was collected and washed extensively with buffer A / 0.5 M NaCl / 40 mM imidazole / 1X protease inhibitor cocktail 3. Subsequently, the resin was incubated with 10ml buffer A / 0.5 M NaCl / 40 mM imidazole / 1X protease inhibitor cocktail 3 / 10 mM $Mg(OAc)_2$ / 2 mM ATP at 4 °C for 10 minutes to remove chaperones, and then washed extensively with buffer A / 0.5 M NaCl / 40 mM imidazole. Proteins were eluted with 5 ml of buffer A / 0.5 M NaCl / 250 mM imidazole. Ulp1 protease (10 μg/ml) was then added to remove this His-SUMO tag from the expressed protein, and the mixture was incubated for 1 h on ice.

The sample was concentrated and loaded onto a 24 ml Superdex 75 column in buffer A / 0.5 M NaCl. The peak fractions were pooled and mixed with 0.5 ml Ni-NTA resin to remove his-tagged Ulp1 and SUMO. The mixture was incubated at 4 °C for 1 h with rotation. The flow-through fraction was collected and dialyzed against buffer A / 0.3 M KOAc at 4 °C overnight. Finally, the dialyzed sample was

recovered, concentrated, aliquoted, snap frozen in liquid nitrogen and stored at – 80 °C.

LET-70

Pellets were resuspended in 20 ml of buffer C / 0.5 M NaCl / 40 mM imidazole / 1X protease inhibitor cocktail 3 with 500 μg / ml Lysozyme, then the mixture was incubated at 4 °C for 0.5 h with rotation. Subsequently, the sample was sonicated twice for 90 s (15 s on, 30 s off) at 40% on a Branson Digital Sonifier. The mixture was centrifuged at 100,000 x g at 4°C for 0.5 h. After spinning, the soluble extract was recovered and mixed with 1 ml Ni-NTA resin (30210, QIAGEN). The mixture was incubated at 4 °C for 2 h with rotation.

Resin was collected and washed extensively with buffer C / 0.5 M NaCl / 40 mM imidazole / 1X protease inhibitor cocktail 3. Then resin was then incubated with 10ml buffer C / 0.5 M NaCl / 40 mM imidazole / 1X protease inhibitor cocktail 3 / 10 mM $Mg(OAc)_2$ / 2 mM ATP at 4 °C for 10 minutes to remove chaperones, then washed extensively with buffer C / 0.5 M NaCl / 40 mM imidazole. Proteins were eluted with 5 ml of buffer C / 0.5 M NaCl / 250 mM imidazole. TEV protease (100 μg) was added into the eluate fractions, and the mixture was incubated at 4 °C overnight with rotation.

The sample was concentrated and loaded onto a 24 ml Superdex 75 column in buffer C / 0.3 M KOAc. Peak fractions containing LET-70 were pooled and mixed with 0.5 ml Ni-NTA resin to remove His-tagged TEV protease and uncleaved His-LET-70. The mixture was incubated at 4 °C for 1 h with rotation. The flow-through fraction was collected and re-loaded onto a 24 ml Superdex 75 column in buffer C /

0.3 M KOAc. Finally, peak fractions were pooled, concentrated, aliquoted and snap frozen in liquid nitrogen and stored at - 80 °C.

MCM-10

Pellets were resuspended in 20 ml of buffer B / 0.5 M NaCl / 40 mM imidazole / 1X protease inhibitor cocktail 3 with 500 μg / ml Lysozyme, then the mixture was incubated at 4 °C for 0.5 h with rotation. Subsequently, the sample was sonicated twice for 90 s (15 s on, 30 s off) at 40% on a Branson Digital Sonifier. The mixture was centrifuged at 100,000 x g at 4°C for 0.5 h. After spinning, the soluble extract was recovered and mixed with 1 ml Ni-NTA resin (30210, QIAGEN). The mixture was incubated at 4 °C for 2 h with rotation.

Resin was collected and washed extensively with buffer B / 0.5 M NaCl / 40 mM imidazole / 1X protease inhibitor cocktail 3. The resin was then incubated with 10ml buffer B / 0.3 M NaCl / 40 mM imidazole / 1X protease inhibitor cocktail 3 / 10 mM $Mg(OAc)_2$ / 2 mM ATP at 4 °C for 10 minutes to remove chaperones, and then washed extensively with buffer B / 0.15 M NaCl / 40 mM imidazole. Proteins were eluted with 5 ml of buffer B / 0.15 M NaCl / 250 mM imidazole. Ulp1 protease (10 μg/ml) was then added and the mixture was incubated for 1 h on ice.

The eluate fractions were diluted to 10 ml and loaded onto a 1 ml HiTrap SP HP column in buffer B / 0.15 M NaCl. MCM-10 was eluted with a 20 ml gradient from 0.15 – 0.4 M NaCl and then 10 ml gradient from 0.4 – 1 M NaCl in buffer B. The peak fractions were pooled, diluted to 0.15 M NaCl and loaded onto a 1 ml HiTrap SP HP column in buffer B / 0.15 M NaCl, MCM-10 was eluted with a 25 ml gradient from 0.15 – 0.4 M NaCl and then 5 ml gradient from 0.4 – 1 M NaCl in buffer B. The peak fractions were re-loaded on 1 ml HiTrap SP HP by using same

procedure. Finally, the peak fractions were pooled, concentrated, aliquoted, snap frozen in liquid nitrogen and stored at – 80 °C.

UBC-1, UBC-3, UBC-7 and UBC-14

Pellets were resuspended in 20 ml of buffer A / 0.4 M NaCl / 40 mM imidazole / 1X protease inhibitor cocktail 3 with 500 μg / ml Lysozyme, then the mixture was incubated at 4 °C for 0.5 h with rotation. Subsequently, the sample was sonicated twice for 90 s (15 s on, 30 s off) at 40% on a Branson Digital Sonifier. The mixture was centrifuged at 100,000 x g at 4°C for 0.5 h. After spinning, the soluble extract was recovered and mixed with 1 ml Ni-NTA resin (30210, QIAGEN). The mixture was incubated at 4°C for 2 h with rotation and the resin collected and washed extensively with buffer A / 0.4 M NaCl / 40 mM imidazole / 1X protease inhibitor cocktail 3. The resin was then incubated with 10ml buffer A / 0.4 M NaCl / 40 mM imidazole / 1X protease inhibitor cocktail 3 / 10 mM $Mg(OAc)_2$ / 2 mM ATP at 4 °C for 10 minutes to remove chaperones, then washed extensively with buffer A / 0.4 M NaCl / 40 mM imidazole. Proteins were eluted with 5 ml of buffer A / 0.4 M NaCl / 250 mM imidazole. TEV protease (100 μg) was added to the eluate fractions, and the mixtures were incubated at 4°C overnight with rotation.

The samples were diluted to 0.2 M NaCl and loaded onto a 1 ml HiTrap Q column in buffer A / 0.2 M NaCl. The expressed proteins were eluted with a 20 ml gradient from 0.2 – 1 M NaCl in buffer A. The peak fractions were then pooled and mixed with 0.5 ml Ni-NTA resin to remove his-tagged TEV protease and uncleaved target protein. The mixture was incubated at 4 °C for 1 h with rotation. The flow-through fraction was collected and dialyzed against buffer A / 0.3 M KOAc at 4 °C

overnight. Finally, the dialyzed sample was recovered, aliquoted, snap frozen in liquid nitrogen and stored at -80 °C.

UBC-12

Pellets were resuspended in 20 ml of buffer D / 0.5 M NaCl / 40 mM imidazole / 1X protease inhibitor cocktail 3 with 500 μg / ml Lysozyme, then the mixture was incubated at 4 °C for 0.5 h with rotation. Subsequently, the sample was sonicated twice for 90 s (15 s on, 30 s off) at 40% on a Branson Digital Sonifier. The mixture was centrifuged at 100,000 x g at 4°C for 0.5 h. After spinning, the soluble extract was recovered and mixed with 1 ml Ni-NTA resin (30210, QIAGEN). The mixture was incubated at 4 °C for 2 h with rotation.

Resin was collected and washed extensively with buffer D / 0.5 M NaCl / 40 mM imidazole / 1X protease inhibitor cocktail 3. The resin was then incubated with 10ml buffer D / 0.5 M NaCl / 40 mM imidazole / 1X protease inhibitor cocktail 3 / 10 mM $Mg(OAc)_2$ / 2 mM ATP at 4 °C for 10 minutes to remove chaperones, and then washed extensively with buffer D / 0.5 M NaCl / 40 mM imidazole. Proteins were eluted with 5 ml of buffer D / 0.5 M NaCl / 250 mM imidazole. Ulp1 protease (10 µg/ml) was added to cleave the His-SUMO tag from UBC-12 and the mixture was incubated for 1 h on ice.

The sample was concentrated and loaded onto a 24 ml Superdex 75 column in buffer D / 0.5 M NaCl. The peak fractions were pooled and mixed with 0.5 ml Ni-NTA resin to remove his-tagged Ulp1 and SUMO. The mixture was incubated at 4 °C for 1 h with rotation. The flow-through fraction was collected and loaded onto a 24 ml Superdex 75 column in buffer D / 0.5 M NaCl. Finally, the peak fractions were pooled, concentrated, aliquoted, snap frozen in liquid nitrogen and stored at - 80 °C.

UBC-18 and NED-8

Pellets were resuspended in 20 ml of buffer C / 0.5 M NaCl / 40 mM imidazole / 1X protease inhibitor cocktail 3 with 500 μg / ml Lysozyme, then the mixture was incubated at 4 °C for 0.5 h with rotation. Subsequently, the sample was sonicated twice for 90 s (15 s on, 30 s off) at 40% on a Branson Digital Sonifier. The mixture was centrifuged at 100,000 x g at 4°C for 0.5 h. After spinning, the soluble extract was recovered and mixed with 1 ml Ni-NTA resin (30210, QIAGEN). The mixture was incubated at 4 °C for 2 h with rotation.

Resin was collected and washed extensively with buffer C / 0.5 M NaCl / 40 mM imidazole / 1X protease inhibitor cocktail 3. The resin was then incubated with 10ml buffer C / 0.5 M NaCl / 40 mM imidazole / 1X protease inhibitor cocktail 3 / 10 mM $Mg(OAc)_2$ / 2 mM ATP at 4 °C for 10 minutes to remove chaperones and then washed extensively with buffer C / 0.5 M NaCl / 40 mM imidazole. Proteins were eluted with 5 ml of buffer C / 0.5 M NaCl / 250 mM imidazole. Subsequently, Ulp1 protease (10 μg/ml) was added to cleave the HIS_{14}-SUMO tag from the expressed protein, and the mixture was incubated for 1 h on ice.

Subsequently, the sample was concentrated and loaded onto a 24 ml Superdex 75 column in buffer C / 0.5 M NaCl. Peak fractions were pooled and mixed with 0.5 ml Ni-NTA resin to remove his-tagged Ulp1 protease and SUMO. The mixture was incubated at 4 °C for 1 h with rotation. The flow-through fraction was collected and dialyzed against buffer C / 0.5 M NaCl (for UBC-18) or 0.3 M KOAc (for NED-8) at 4 °C overnight. Finally, the dialyzed sample was recovered, concentrated, aliquoted, snap frozen in liquid nitrogen and stored at – 80 °C.

UBXN-3 (full-length) and UBXN-3-ΔUBX (amino acids 1-530)

Rosetta cells were transformed with expression vectors for full-length UBXN-3 or UBXN-3-ΔUBX. For induction, 1 mM IPTG was added and cells were incubated at 24 °C (full-length UBXN-3) or 18°C (UBXN-3-ΔUBX) for 16 h. The cell pellets were resuspended in 20 ml of buffer H / 0.5 M NaCl / 30 mM imidazole / 5 mM $Mg(OAc)_2$ / 1X protease inhibitor cocktail 3 with 500 μg / ml Lysozyme, then the mixture was incubated at 4 °C for 0.5 h with rotation. Subsequently, the sample was sonicated twice for 90 s (15 s on, 30 s off) at 40% on a Branson Digital Sonifier. The mixture was centrifuged at 100,000 x g at 4°C for 0.5 h. After spinning, the soluble extract was recovered and mixed with 2 ml Ni-NTA resin (30210, QIAGEN). The mixture was incubated at 4 °C for 2 h with rotation.

Resin was collected and washed extensively with buffer H / 0.5 M NaCl / 30 mM imidazole / 5 mM $Mg(OAc)_2$. Proteins were eluted with 6 ml of buffer H / 0.5 M NaCl / 0.5 M imidazole / 5 mM $Mg(OAc)_2$. Subsequently, Ulp1 protease (10 μg/ml) was added and the mixture was incubated for 1 h on ice. The sample was then concentrated to 500 μl and loaded onto a 24 ml Superose 6 column in 20 mM Hepes-KOH (pH 7.4), 0.3 M sorbitol, 0.15 M NaCl, 5 mM $Mg(OAc)_2$, 0.5 mM TCEP. Finally, UBXN-3-containing fractions were pooled, concentrated, aliquoted, snap frozen in liquid nitrogen and stored at – 80 °C.

UFD-1_NPL-4.1

Untagged NPL-4.1 (isoform a) and HIS_{14}-SUMO-UFD-1 were expressed separately. Rosetta cells were transformed with expression vectors for 14HIS-SUMO-UFD-1 or NPL-4.1 expression vectors. Subsequently, transformant colonies were grown in parallel, before addition of 1 mM IPTG and expression at 18 °C for 11

h. Bacterial pellets were mixed at a 3:1 ratio (NPL-4.1: 14HIS-SUMO-UFD-1) and resuspended in 20 ml of buffer H / 0.5 M NaCl / 30 mM imidazole / 1X protease inhibitor cocktail 3 with 500 μg / ml Lysozyme, then the mixture was incubated at 4 °C for 0.5 h with rotation. Subsequently, the sample was sonicated twice for 90 s (15 s on, 30 s off) at 40% on a Branson Digital Sonifier. The mixture was centrifuged at 100,000 x g at 4°C for 0.5 h. After spinning, the soluble extract was recovered and mixed with 1.5 ml Ni-NTA resin (30210, QIAGEN). The mixture was incubated at 4 °C for 2 h with rotation.

Resin was collected and washed extensively with buffer H / 0.5 M NaCl / 5 mM Mg(OAc)2 / / 30 mM imidazole. The resin was further washed with 10ml buffer H / 0.5 M NaCl / 60 mM imidazole / 10 mM Mg(OAc)2 / 2 mM ATP at 4 °C for 10 minutes to remove chaperones. Proteins were eluted with 3.75 ml of buffer H / 0.5 M NaCl / 5mM Mg(OAc)2 / 0.4 M imidazole. Subsequently, Ulp1 protease (10 μg/ml) was added and the mixture was dialyzed into buffer H / 0.5 M NaCl / 5 mM $Mg(OAc)_2$ / 30 mM imidazole at 4 °C for 4 h. The dialyzed mixture was mixed with 1 ml Ni-NTA resin to remove His-tagged Ulp1 and SUMO. The mixture was incubated at 4 °C for 1 h with rotation. The flow-through fraction was collected and then concentrated to 500 μl and loaded onto a 24 ml Superdex 200 column in 20 mM Hepes-KOH (pH 7.8), 0.3 M sorbitol, 0.15 M NaCl, 5 mM $Mg(OAc)_2$, 0.5 mM TCEP. Finally, UFD-1_NPL-4.1 containing fractions were pooled, concentrated, aliquoted, snap frozen in liquid nitrogen and stored at – 80 °C.

ULP1 catalytic domain (amino acids 403-621)

A purified version of Ulp1 403-621 was initially provided by Dr. Alexander Stein, Max Plank Institute for Biophysical Chemistry, Gottingen, Germany. Subsequently, the protein was expressed and purified as follows:

A 1-litre culture was used for this purification. Around 5 ml volume of cell pellet were resuspended in 20 ml of buffer H / 0.5 M NaCl / 5 mM $Mg(OAc)_2$ / 20 mM imidazole / 10% glycerol / 0.01% IGEPAL CA-630 / 1X protease inhibitor cocktail 3 with 500 μg / ml Lysozyme, then the mixture was incubated at 4 °C for 0.5 h with rotation. Subsequently, the sample was sonicated twice for 90 s (15 s on, 30 s off) at 40% on a Branson Digital Sonifier. The mixture was centrifuged at 100,000 x g at 4°C for 0.5 h. After spinning, the soluble extract was recovered and mixed with 3 ml Ni-NTA resin (30210, QIAGEN). The mixture was incubated at 4 °C for 2 h with rotation.

Resin was collected and washed extensively with 150 ml of buffer H / 0.5 M NaCl / 5 mM $Mg(OAc)_2$ / 20 mM imidazole / 10% glycerol / 0.01% IGEPAL CA-630 / 1X protease inhibitor cocktail 3. Proteins were eluted with 10 ml of 20 ml of buffer H / 0.5 M NaCl / 5 mM $Mg(OAc)_2$ / 250 mM imidazole / 10% glycerol / 0.01% IGEPAL CA-630 / 1X protease inhibitor cocktail 3. The sample was concentrated and loaded onto a 24 ml Superdex 75 column in buffer H / 0.5 M NaCl / 5 mM $Mg(OAc)_2$ / 10% glycerol / 0.01% IGEPAL CA-630. The peak fractions were pooled, concentrated, aliquoted, snap frozen in liquid nitrogen and stored at - 80 °C.
