# Supplementary material for: TIMELESS‐TIPIN and UBXN‐3 promote replisome disassembly during DNA replication termination in Caenorhabditis elegans
Source: EMBO J. 2021 Jul 16;40(17):e108053. doi: 10.15252/embj.2021108053 (PMC8408604; doi:10.15252/embj.2021108053)
Supplement: Supplementary file 3 — Expanded View Figures PDF [file EMBJ-40-e108053-s002.pdf]

Expanded View Figures

Figure EV1. The role of *C. elegans* LET-70, ARI-1_UBC-18 and cullin neddylation in reconstituted CMG-MCM-7 ubiquitylation.

A, B Reconstituted CMG-MCM-7 ubiquitylation reactions were performed as in Fig 2, in the presence of the indicated factors. "Neddylation" indicates addition of the *C. elegans* ULA-1_RFL-1 E1 enzyme, the UBC-12 E2 enzyme, the DCN-1 E3 enzyme and NED-8.

C Similar reactions were performed in the presence of the indicated E2 enzymes and ubiquitin variants.

D Comparison of purified CUL-2^{LRR-1} containing either wt CUL-2 or CUL-2-2R. The latter has mutation of lysine 719 and lysine K479, which in human CUL-2 comprise a neddylation site and a site of interaction with the DCN-1 E3 ligase for neddylation (Bandau *et al*, 2012). We previously showed that the combined mutation of these two sites prevents human CUL-2 from supporting CMG-MCM-7 ubiquitylation in *Xenopus* egg extracts (Sonneville *et al*, 2017).

E The ability of CUL-2^{LRR-1}, CUL-2-2R^{LRR-1} and CUL-2^{VHL-1} to support the formation of free ubiquitin chains by UBC-3 was monitored in reactions containing FLAG-tagged ubiquitin and the indicated factors. CUL-2 neddylation and the formation of ubiquitin chains was then monitored by immunoblotting with anti-CUL-2 or anti-FLAG antibodies.

F CMG-MCM-7 ubiquitylation reactions were performed as above with the indicated factors.

Data information: Wt = wild-type ubiquitin; K0 = all lysines mutated to arginine; K48R = lysine 48 mutated to arginine; K48-only = all lysines of ubiquitin mutated to arginine except for lysine 48.

© 2021 The Authors

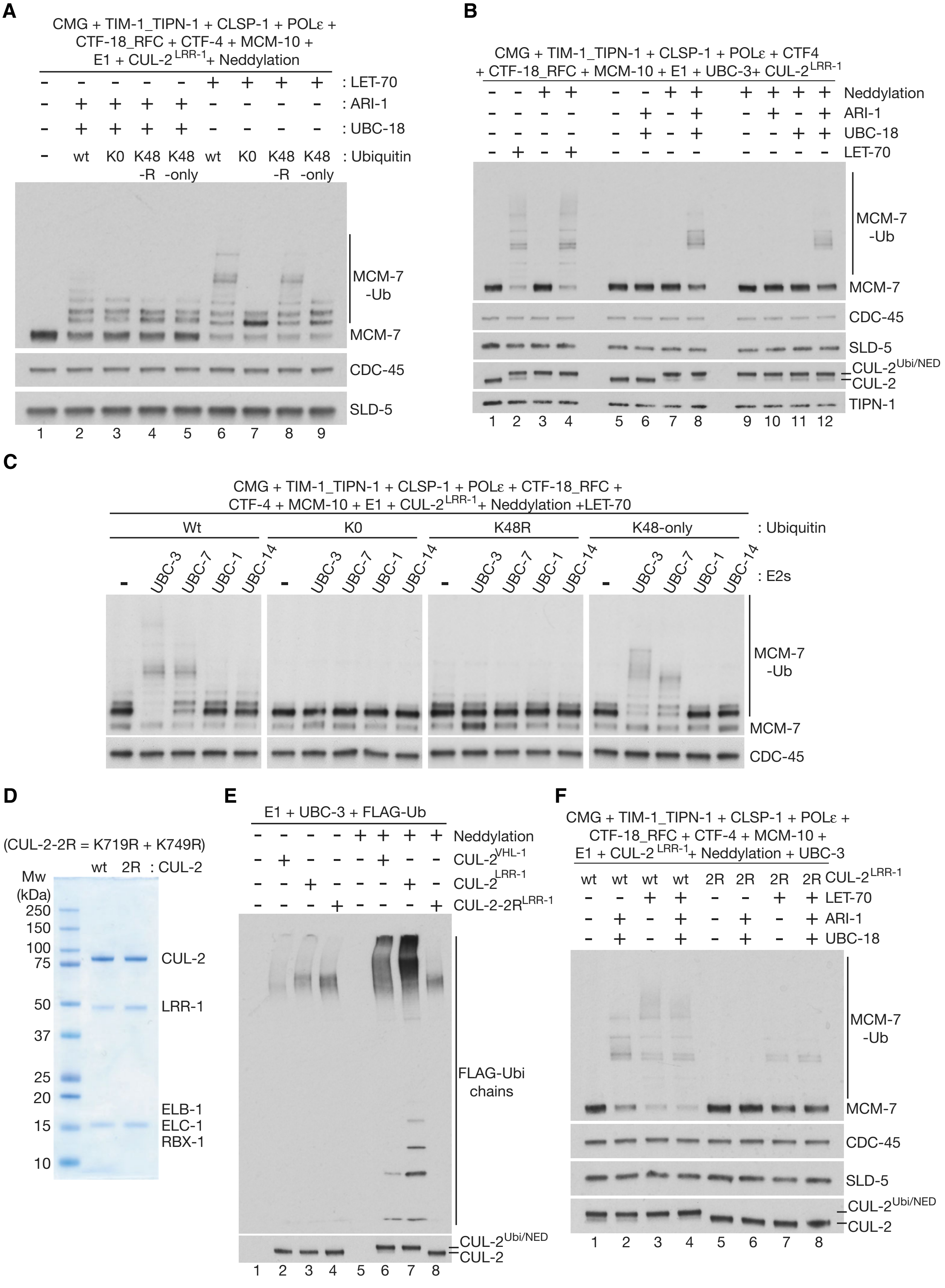

Figure EV1.

 © 2021 The Authors

Figure EV2. *C. elegans* TIM-1_TIPN-1 promotes the priming of CMG-MCM-7 ubiquitylation by LET-70 and ARI-1_UBC-18.

A Purified CMG, TIM-1_TIPN-1, POLε, CLSP-1, CTF-18_RFC, CTF-4, MCM-10 and CUL-2^{LRR-1} were mixed before immunoprecipitation of CMG using polyclonal antibodies to SLD-5. The association of CMG with other replisome factors was then monitored by immunoblotting.

B Three repeats of the experiment in Fig 3B.

C Quantification of the data in (B). For each sample, the proportion of modified MCM-7 was calculated, relative to unmodified MCM-7. The panel shows the mean values from the three repeats, together with the standard deviation.

D Reconstituted CMG-MCM-7 ubiquitylation reactions were performed as in Fig 3, in the presence of the indicated factors (including 15 nM CUL-2^{LRR-1}). “Neddylation” indicates addition of the *C. elegans* ULA-1_RFL-1 E1 enzyme, the UBC-12 E2 enzyme, the DCN-1 E3 enzyme and NED-8.

E Similar reactions were performed in the presence of 3 nM CUL-2^{LRR-1}, to reveal the contribution of TIM-1_TIPN-1 to priming of CMG-MCM-7 ubiquitylation by LET-70.

F Glycerol gradient analysis of the indicated protein mixtures, in the presence of annealed oligonucleotides with 45 bp double-strand DNA and 39nt single-strand 3’ flap DNA (see Materials and Methods and Appendix Table S1).

G In reactions analogous to those in Fig 3E and F, ubiquitylation of the indicated factors was monitored by immunoblotting. Reactions were performed in sets of three as indicated (1 = dropout of CUL-2^{LRR-1}; 2 = wt ubiquitin; 3 = lysine-free or K0 ubiquitin).

H Ubiquitylation of the TIM-1_TIPN-1 complex was monitored in the presence or absence of the indicated factors.

© 2021 The Authors

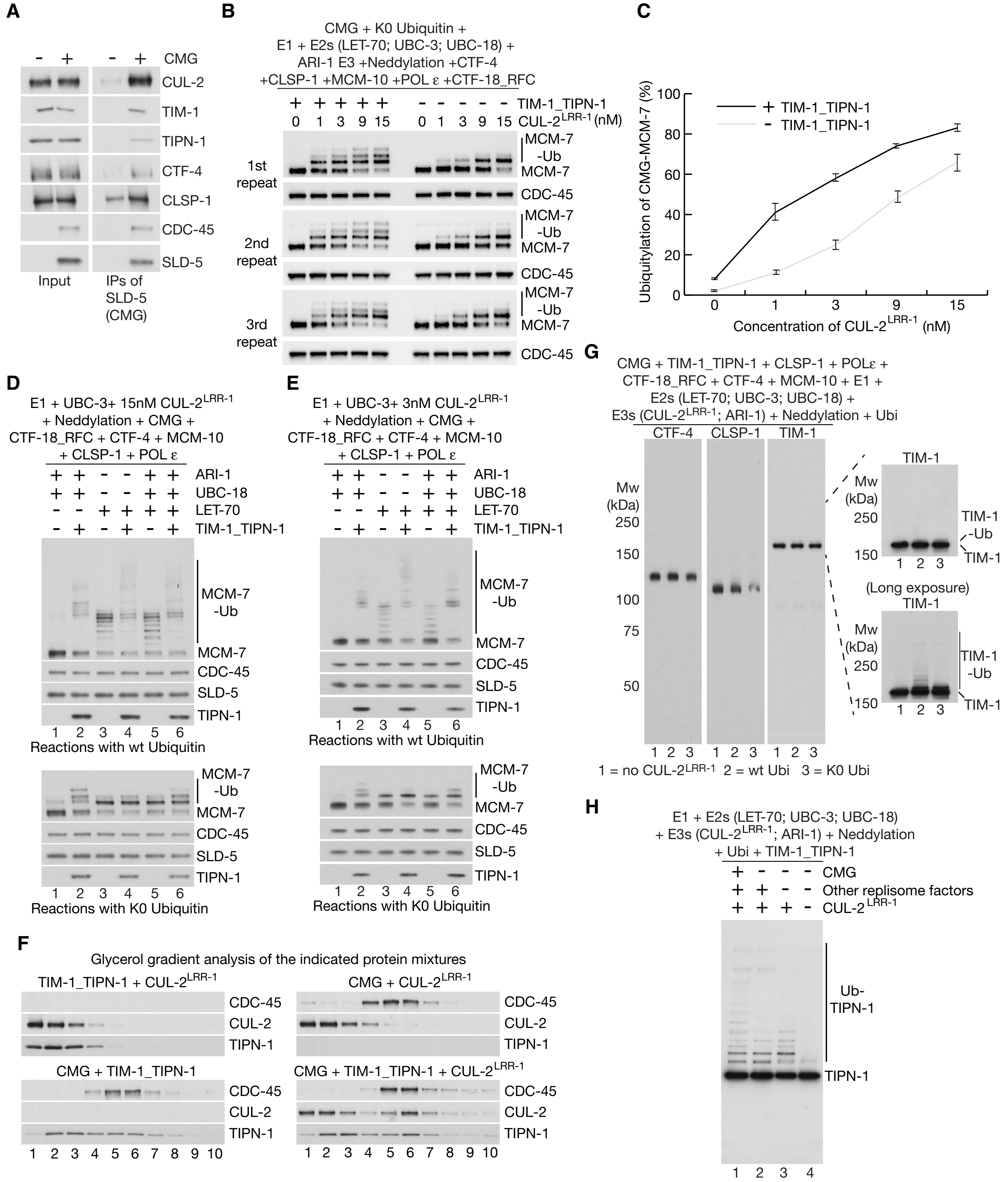

Figure EV2.

 © 2021 The Authors

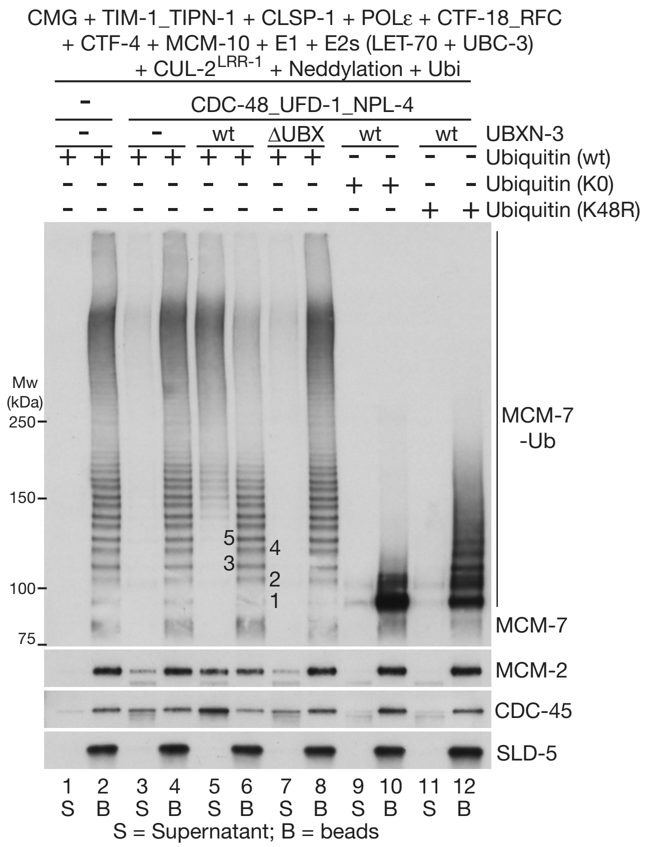

Figure EV3. ***C. elegans* CMG disassembly is dependent upon polyubiquitylation and the UBX domain of UBXN-3.**

Reactions were performed as described for Fig 4, in the presence of the indicated factors. "Neddylation" indicates addition of the *C. elegans* ULA-1_RFL-1 E1 enzyme, the UBC-12 E2 enzyme, the DCN-1 E3 enzyme and NED-8. The bands corresponding to MCM-7 conjugated to 1–5 ubiquitins are indicated in lane 6.

© 2021 The Authors

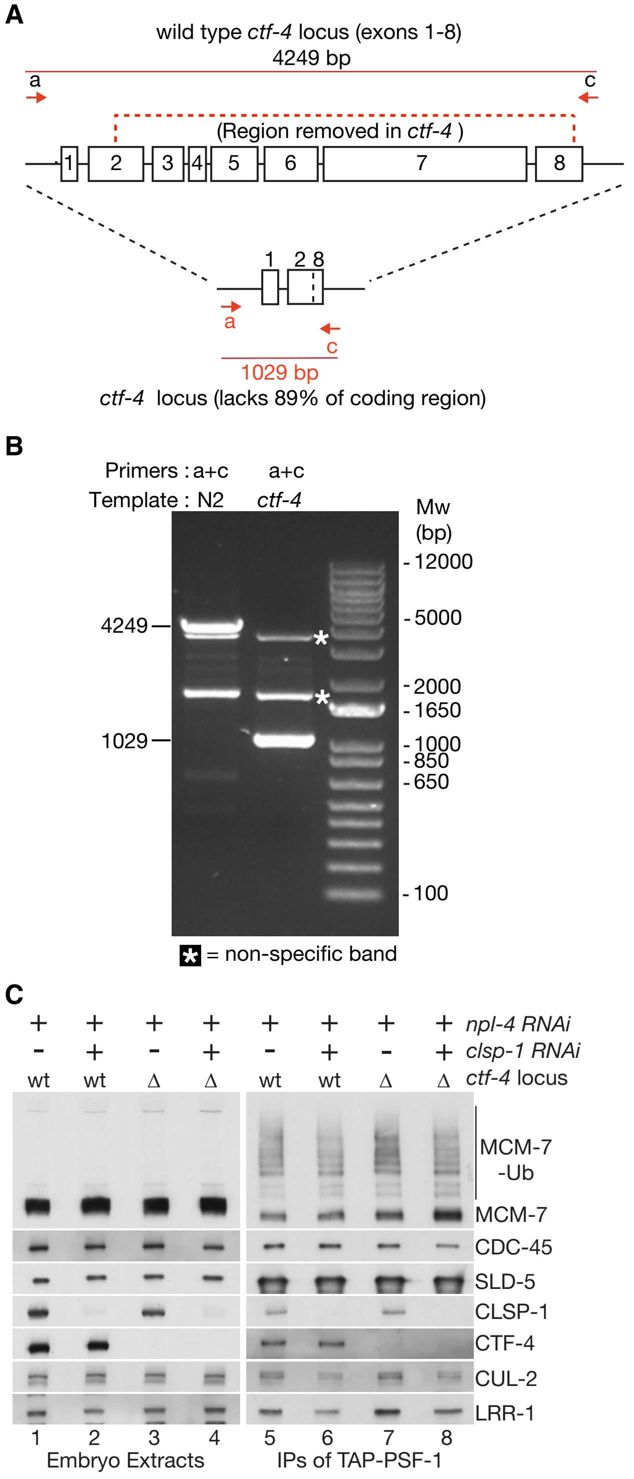

Figure EV4. Deletion of the *C. elegans ctf-4* gene and RNAi depletion of CSLP-1 does not impair the *in vivo* ubiquitylation of CMG-MCM-7 during DNA replication termination.

A Illustration of the region of the *C. elegans ctf-4* gene that was deleted by CRISPR-Cas9. Arrows indicate PCR primers that were used to monitor the deletion in adult worms.

B PCR analysis of wt and *ctf-4*Δ worms with the indicated primers (non-specific bands are denoted with asterisks).

C Control or *ctf-4*Δ worms were subjected to RNAi to *npl-4* and *clsp-1* as indicated, before isolation of TAP-PSF-1 from cell extracts. The association of the indicated factors with TAP-PSF-1 was monitored by immunoblotting, together with ubiquitylation of CMG-MCM-7.

 © 2021 The Authors

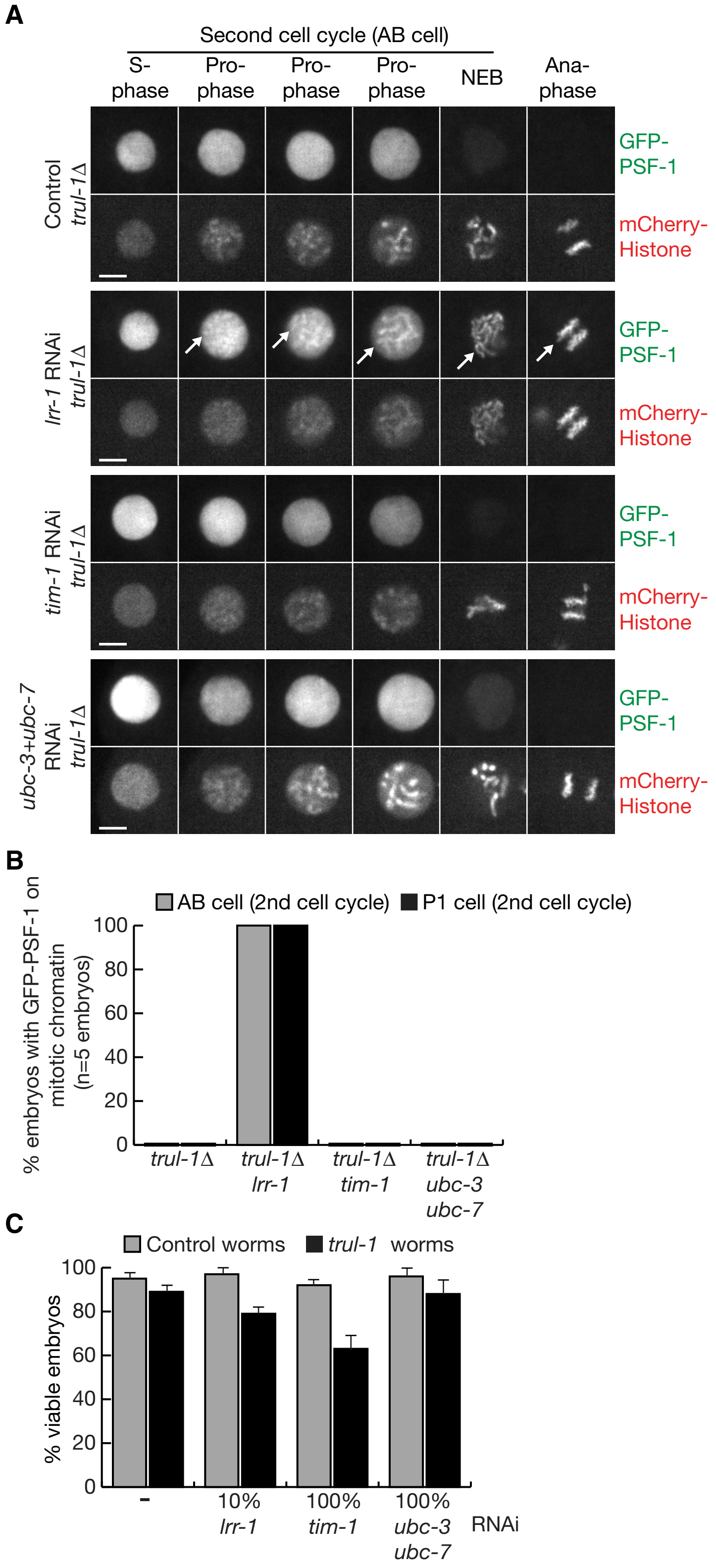

Figure EV5. RNAi inactivation of *tim-1* in *trul-1*Δ worms does not lead to persistence of CMG on mitotic chromatin.

A The presence of GFP-PSF-1 on mitotic chromatin (indicated by white arrows) was monitored by spinning disc confocal microscopy (see Materials and Methods), in *trul-1*Δ worms exposed to the indicated RNAi treatments ("Control" = worms fed on bacteria containing empty vector). The scale bars correspond to 5 μm.

B Quantification of the data in (A).

C Embryonic viability was measured as above for the indicated RNAi treatments of wild-type (control) or *trul-1*Δ worms. The data represent the means and standard deviations from three biological replicates.

© 2021 The Authors
